# Childhood obesity’s effect on cognition and brain connectivity worsens with low family income

**DOI:** 10.1172/jci.insight.181690

**Published:** 2024-07-09

**Authors:** Dardo Tomasi, Nora D. Volkow

**Affiliations:** National Institute on Alcohol Abuse and Alcoholism, Bethesda, Maryland, USA.

**Keywords:** Development, Neuroscience, Obesity

## Abstract

Childhood obesity and its adverse health consequences have risen worldwide, with low socioeconomic status increasing the risk in high-income countries like the United States. Understanding the interplay between childhood obesity, cognition, socioeconomic factors, and the brain is crucial for prevention and treatment. Using data from the Adolescent Brain Cognitive Development (ABCD) study, we investigated how body mass index (BMI) relates to brain structural and functional connectivity metrics. Children with obesity or who are overweight (*n* = 2,356) were more likely to live in poverty and exhibited lower cognitive performance compared with children with a healthy weight (*n* = 4,754). Higher BMI was associated with multiple brain measures that were strongest for lower longitudinal diffusivity in corpus callosum; increased activity in cerebellum, insula, and somatomotor cortex; and decreased functional connectivity in multimodal brain areas, with effects more pronounced among children from low-income families. Notably, nearly 80% of the association of low income and 70% of the association of impaired cognition on BMI were mediated by higher brain activity in somatomotor areas. Increased resting activity in somatomotor areas and decreased structural and functional connectivity likely contribute to the higher risk of being overweight or having obesity among children from low-income families. Supporting low-income families and implementing educational interventions to improve cognition may promote healthy brain function and reduce the risk of obesity.

## Abstract 2

Childhood obesity, linked to low socioeconomic status, correlates with altered brain activity and connectivity, especially in low-income families. Interventions targeting cognition and income could mitigate obesity risk.

## Introduction

Pediatric obesity has been on the rise worldwide, and current estimates indicate that 19.7% of children are obese in the United States (1). Several factors contribute to pediatric obesity, including low family income (2), genetics (3), and environmental factors such as high-calorie foods and lack of open spaces for exercising (4). Because childhood obesity can interfere with normal brain development (5–8) and has been associated with diminished cognitive function (7, 9, 10), it is urgent to better understand how it influences brain function. The brain matures rapidly in early childhood, achieving approximately 90% of its adult size by age 6. Additionally, as children progress through late childhood and adolescence, white matter (WM) volume and the fractional anisotropy (FA) of the WM fiber bundles generally increase, whereas gray matter (GM) volume decreases and brain functional connectivity reorganizes, decreasing in some networks while increasing in others (11–13). While the relationship between body mass index (BMI) and brain structural and functional connectivity is complex and potentially bidirectional, the exact mechanisms and the extent to which BMI is associated with alterations in structural and functional connectivity during brain development remain poorly understood. Furthermore, because poverty also negatively affects brain development and cognitive function (14–18), it is also relevant to assess the effect of family income on the relationship between BMI and brain connectivity and cognition. A better understanding of the complex interplay between BMI and brain development could help guide prevention and treatment strategies for childhood obesity (19).

Here we measured the associations between BMI and brain activity, connectivity (structural and functional), and cognitive performance and assessed the influence of family income on these associations. We hypothesized a bidirectional association between BMI and brain structure, function, and cognitive performance, which may be exacerbated among children from low-income families. For this purpose, we used the baseline data from the Adolescent Brain Cognitive Development (ABCD) study, which was collected when children were 9–10 years old and divided the sample into Discovery and Replication subsamples, to assess the reproducibility of the findings.

To measure the structural connectivity provided by WM fibers, we used diffusion tensor imaging (DTI) metrics, including FA, mean diffusivity (MD), longitudinal or axial diffusivity (lD), and radial diffusivity (rD) (8). To measure resting brain activity, we mapped the fractional amplitude of low-frequency fluctuations (fALFF), which measures spontaneous brain activity (20). To quantify resting functional connectivity, we mapped global functional connectivity density (gFCD) (21), a measure that is sensitive to both cognitive performance (22) and family income (15). To assess cognition, we used the composite scores of crystallized, fluid, and total intelligence (23). Given the associations of BMI with WM integrity previously observed in children (8, 14), we specifically hypothesized that higher BMI would be associated with lower cognitive performance, higher resting brain activity in primary cortical areas, and weaker structural and functional connectivity and that these effects would be exacerbated in children from low-income families. To address the complexity of these relationships, we employed causal mediation analysis (CMA) to examine the hypothesized bidirectional associations. In our models, we treated BMI as both an independent and dependent variable, enabling us to test the directionality of its effects on brain and cognitive outcomes. We included family income as a mediator to explore how socioeconomic status influences these relationships.

## Results

### Demographic characteristics.

Among the 7,110 children in the present study, 4,754 were healthy weight (67%) and 2,356 overweight or obese (33%) based on the 85th percentile for BMI according to age and sex. This classification method is standard in pediatric studies, as it accounts for the variations in BMI that occur naturally with growth and development (24). Therefore, overweight and obesity in children are not defined using a fixed BMI threshold (such as BMI > 30, which is used for adults). These proportions align with the reported rate of being overweight or having obesity (35.4%) among American children and adolescents aged 2–19 years in the 2017–2018 period (25), corresponding to the time frame when the ABCD baseline data were collected. Analysis of racial and ethnic differences revealed a higher percentage of children with obesity or who are overweight from families that identified as African American (30% and 18%) and Hispanic (25% and 19%) compared with families that identified as White (10% and 14%). Analyses of income differences revealed that children with obesity or who are overweight were more likely to reside in poverty (defined in the United States as a family income below $25,000) than children in a healthy weight range, with odds ratios of 0.20 and 0.15 for children with obesity or who are overweight, respectively, compared with 0.09 for children in a healthy weight range. Children with obesity or who are overweight were more likely to show depression symptoms than children in a healthy weight range (6.1% versus 3.3%; df = 1, *n* = 4,422, χ^2^ = 16.4, *P* = 3.3 × 10^–5^).

### Discovery and Replication subsamples.

The proportions of children of healthy weight and children with obesity or who are overweight did not differ between the Discovery and Replication samples (χ^2^ = 2.3; *P* = 0.13). There were minimal differences in brain volume, sex, and the proportion of MRI manufacturers between the Discovery and Replication subsamples (Table 1).

### Reproducibility of BMI, demographics, and cognition by weight category.

**Figure 1:**
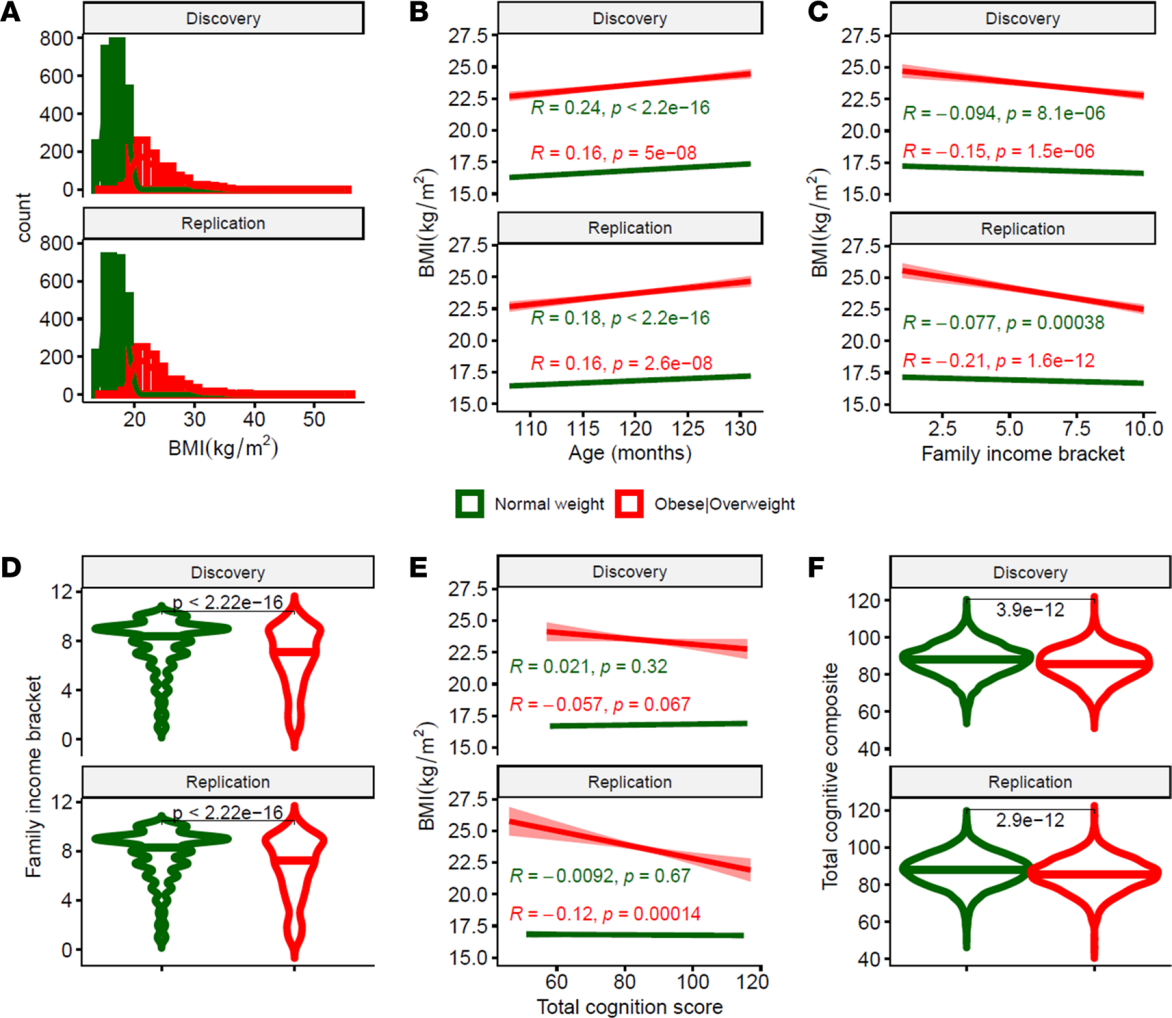
BMI, age, family income, and cognition. (**A** and **B**) Distribution of BMI (**A**) and its age-related increases (**B**) among 3,696 boys and 3,414 girls (4,754 children in a healthy weight range and 2,356 children with obesity or who are overweight) and their reproducibility in Discovery (*n* = 3,597) and Replication (*n* = 3,513) subsamples. (**C** and **E**) In children with obesity or who are overweight, higher BMI was reproducibly linked to lower family income (**C**) and total cognition scores (**E**). (**D** and **F**) Compared with children in a healthy weight range, children with obesity or who are overweight were more likely to reside in lower-income families (**D**) and have lower performance on cognitive tasks (**F**) independently in Discovery and Replication subsamples. BMI percentiles based on age and sex were used to determine weight categories. Numeric labels are 2-sided *P* values reflecting Person correlation analysis (R; **B**, **C**, and **E**) or *t* tests (**D** and **F**).

BMI displayed consistent right-skewed distributions (skewness = 1.5, kurtosis = 4.0) in both the Discovery and Replication data sets that were explained by the weight categories (Figure 1A). There were no differences in BMI between sexes (Supplemental Figure 1; supplemental material available online with this article; https://doi.org/10.1172/jci.insight.181690DS1). Children with healthy weight and children with obesity or who are overweight showed similar increases in BMI with age (Figure 1B). BMI showed a negative correlation with family income bracket, such that higher BMI was associated to lower family income, which was significant independently in the obese/overweight and the healthy weight groups both for the Discovery and Replication samples (Figure 1, C and D). However, note that in the healthy weight group the correlations for the discovery (*r* = –0.094) and replication (*r* = –0.077) samples were very small (Figure 1, C and D).

Cognitive scores differed between groups, being lower for children with obesity or who are overweight than for children in a healthy weight range both for the Discovery and Replication samples (Figure 1F). Among children with obesity or who are overweight, BMI was negatively correlated with total cognitive composite scores, such that higher BMI was associated with lower scores, whereas the correlation was not significant among children in a healthy weight range (Figure 1E), suggesting a threshold effect at which BMI negatively affects cognition. The main effects on BMI of weight category, family income bracket, and total cognitive composite and the interaction between weight category and family income was significant, independently in Discovery and Replication subsamples (*P* < 7 × 10^–5^; F(1,3240)>15.8; ANCOVA; Supplemental Table 1).

### Associations of age, BMI ,and family income with structural connectivity.

**Figure 2:**
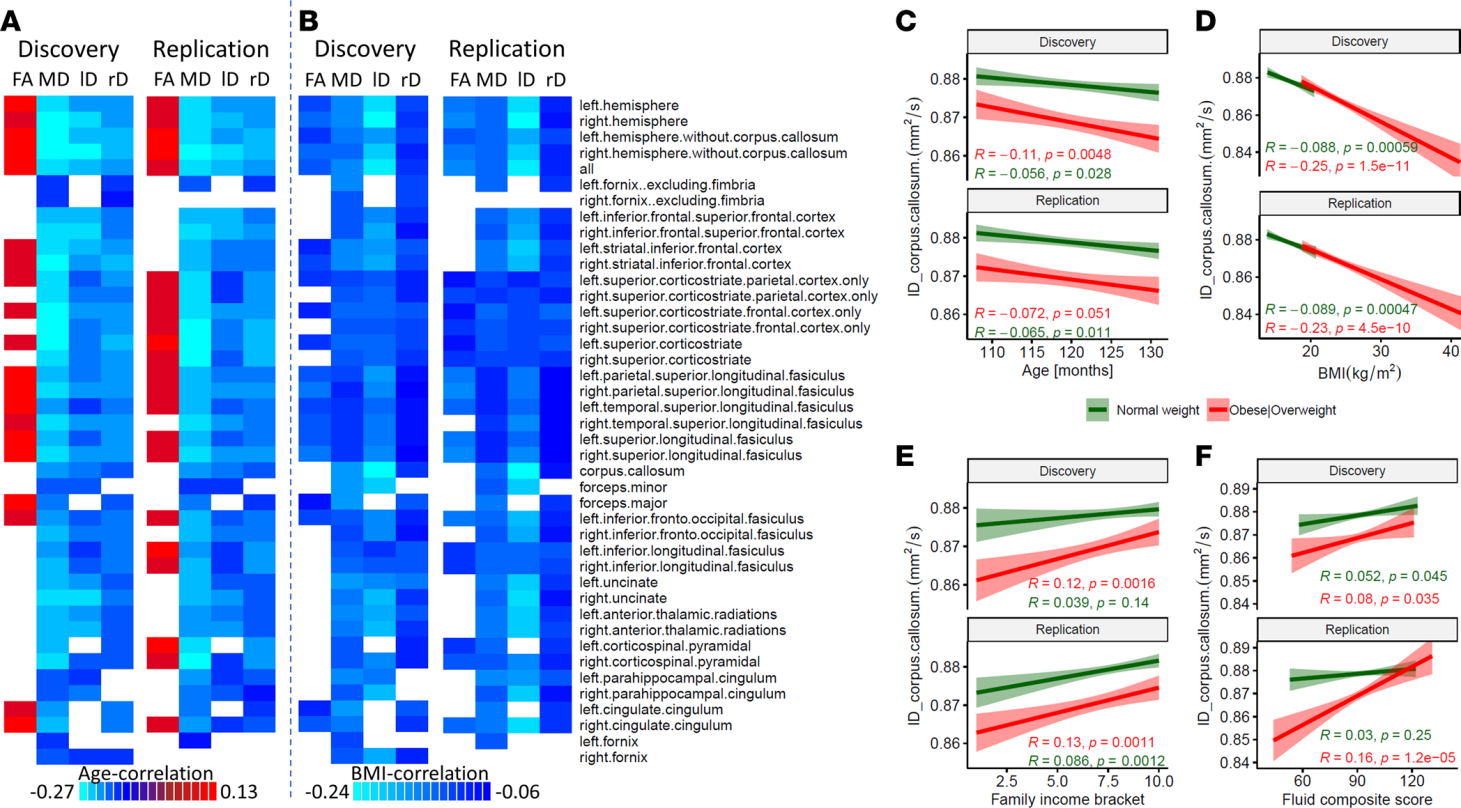
Associations with BMI and age in white matter diffusion. (**A** and **B**) Correlations with age (**A**) and BMI (**B**) for brain volume–corrected fractional anisotropy (FA) and mean (MD), longitudinal (lD) and radial (rD) diffusivities in 42 major white matter fiber bundles across Discovery (*n* = 2,386; 1,625 healthy weight and 761 obese/overweight) and Replication (*n* = 2,411; 1,609 healthy weight and 802 obese/overweight) subsamples. (**C**–**F**) Linear associations of lD in corpus callosum with age (**C**), BMI (**D**), family income bracket (**E**), and fluid cognitive composite score (**F**). Only data collected in Siemens MRI scanners were used for this analysis. The statistical analysis employed an ANCOVA model with a FDR corrected threshold pFDR < 0.05. BMI percentiles based on age and sex were used to determine weight categories.

We used ANCOVA to investigate the associations of age and BMI on WM integrity. The model included 2 weight categories/groups (overweight/obese and healthy weight), 2 independent variables (age and BMI), and a dependent variable (FA, MD, lD, or rD). Independent ANCOVAs were conducted for each DTI metric and ROI. Our analysis revealed negative associations of MD with age and lD with BMI and positive association of age with FA (Figure 2, A and B). Though older age was linked to higher FA and lower MD, lD, and rD (Figure 2, A and C); age-related changes were more notorious for MD than for FA, lD, and rD, particularly in superior cortico-striatal fibers.

Elevated BMI was linked to reduced age-corrected lD across many fiber bundles, notably pronounced in the corpus callosum (*R*[4,796] = –0.24; *P* < 2 × 10^–16^; Cohen’s d = 0.49; Figure 2B), forceps minor, uncinate, anterior thalamic radiations, parahippocampal-cingulum, and cingulate-cingulum fiber bundles (*R*[4,796] < –0.15; *P* < 2 × 10^–16^). The negative correlations in parietal and frontal cortico-striatal bundles were not consistently reproducible. Higher BMI was consistently associated with decreased age-corrected MD and rD bilaterally in various regions, including the uncinate and striatal inferior frontal, cingulum-cingulum fiber bundles, corpus callosum, forceps minor and major, left fornix, the superior cortico-striatal frontal fiber bundle, and left anterior thalamic radiations (Figure 2B). Additionally, increased BMI was linked to decreased age-corrected FA in several regions, including bilateral fornix, right superior longitudinal fasciculus, right inferior to superior frontal, longitudinal fasciculus to right temporal cortex, and bilateral superior parietal cortices WM fiber bundles.

Separate analyses by weight groups showed that lD in corpus callosum decreased with age in both groups (Figure 2C) and was lower in children with obesity or who are overweight than in children in a healthy weight range (Figure 2D). lD in corpus callosum had reproducible positive associations with family income in both children with obesity or who are overweight and children in a healthy weight range and had reproducible positive associations with fluid composite scores in children with obesity (*R*[404] > 0.11; *P* < 0.045, 2-sided), but the association in children in a healthy weight range was not reproducible (Figure 2, D–F). The negative correlation between BMI and lD in corpus callosum was significantly stronger for children with obesity or who are overweight than for children in a healthy weight range, independently for the Discovery and Replication samples (*Z* > 4, *P* < 1 × 10^–5^; Figure 2D). The main effects of age, BMI, weight category, total cognitive composite, and family income on lD in corpus callosum were significant (F[1,4410] > 9, *P* < 0.003; ANCOVA; Supplemental Table 2).

### Association of BMI with brain activity and functional connectivity.

**Figure 3:**
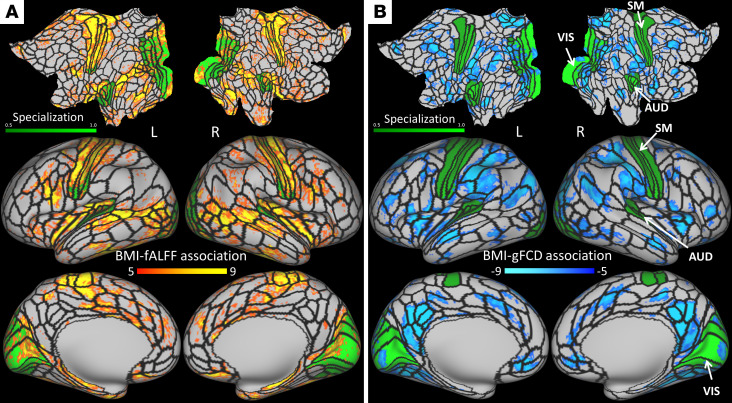
Associations of BMI with fALFF and gFCD. (**A** and **B**) Statistical significance (t-score) for the associations of the BMI with the fractional amplitude of low-frequency fluctuations (fALFF; **A**) and global functional connectivity density (gFCD; **B**) across 7,110 children, and the score of a functional specialization index highlighting unimodal cortical areas (visual, VIS, auditory, AUD, and somatomotor, SM cortices), rendered on flat (top row) and lateral and medial inflated surfaces (middle and bottom rows) of the left (L) and right (R) cerebral hemispheres. Black lines are the contours of 360 multimodal partitions of the human cerebral cortex (27). Significance was found using ANCOVA.

To assess BMI-related differences in resting brain activity and functional connectivity across children, we used fALFF and gFCD metrics, which showed high reproducibility in Discovery and Replication subsamples (Supplemental Figures 2 and 3). Vertex-wise ANCOVA revealed a positive association between BMI and fALFF, which was maximal in cerebellum (Cohen’s d = 0.12) and significant bilaterally in all subcortical regions (Supplemental Figure 4); insular-opercular, somatomotor, and premotor areas; paracentral lobe; orbitofrontal cortex; mid cingulum; early and MT+ visual areas; and lateral and medial temporal cortices (*P*_FDR_ < 0.05; Figure 3A). This pattern exhibited a high level of reproducibility in the Discovery and Replication subsamples (Supplemental Figure 5). The overlap of the BMI-fALFF correlation pattern in the dorsolateral prefrontal, superior, and inferior parietal cortices (including the precuneus) was minimal (~6%; Supplemental Figure 6), suggesting a weak association between BMI and fALFF in these regions. In contrast, higher BMI was associated with lower gFCD, which was maximal in the precuneus (area 7 m; Cohen’s d = 0.18) and also significant bilaterally in other default-mode network (DMN) regions (posterior cingulum, angular gyrus, and medial prefrontal cortex [PFC]); in superior frontal, inferior, and middle temporal gyri; primary and secondary visual areas; inferior and superior parietal cortices; premotor cortex; dorsolateral PFC; frontal pole; and the anterior cerebellar lobe (*P*_FDR_ < 0.05; Figure 3B). These patterns had high reproducibility in the Discovery and Replication subsamples (Supplemental Figure 7).

We applied a functional specialization index (26) that distinguishes between unimodal cortical regions (such as visual, auditory, and somatomotor cortices), characterized by a high specialization index (>0.5), from heteromodal association cortical areas (such as the insula, dorsolateral PFC, and inferior parietal cortex), characterized by a lower specialization index, to map the associations with BMI. The BMI associations with fALFF had a more pronounced overlap with unimodal areas (36%) than the BMI associations with gFCD (11%) (Figure 3).

### Associations of fALFF and gFCD, with cognition, family income, and FA.

**Figure 4:**
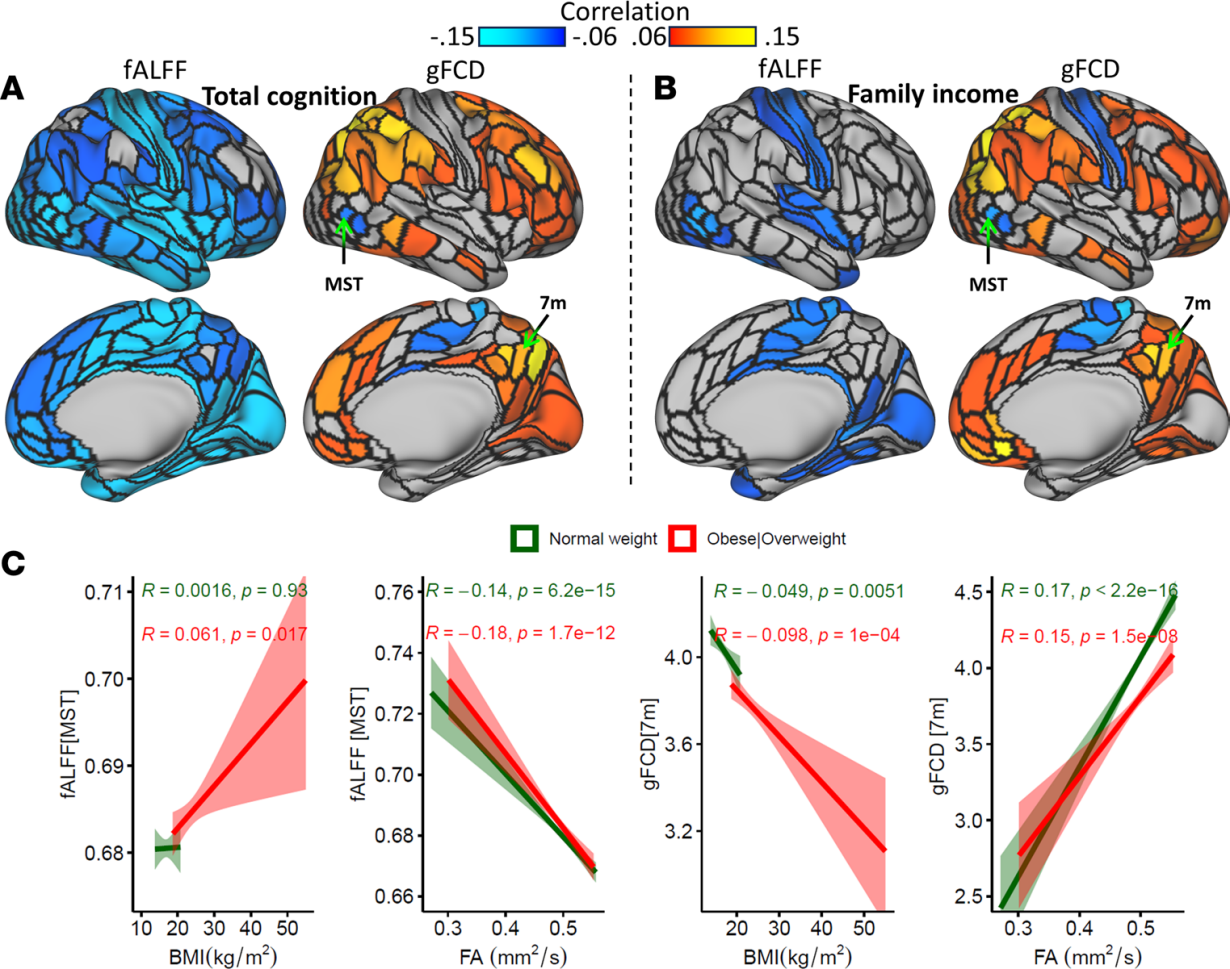
Associations with cognition, income, and fractional anisotropy (FA). (**A** and **B**) In the Discovery subsample, higher cognitive composite score (**A**) or family income bracket (**B**) were associated to lower fractional amplitude of low-frequency fluctuations (fALFF) predominantly in insula, cingulum, lateral visual, and somatomotor cortices; higher global functional connectivity density (gFCD) in frontoparietal and default-mode network regions; and lower gFCD in somatomotor cortex and lateral occipital areas. (**C**) Higher FA, averaged across all white matter fibers in the brain, was associated with lower fractional amplitude of low-frequency fluctuations (fALFF) in the medial superior temporal (MST) area and with higher global functional connectivity density (gFCD) in precuneus (the medial part of Brodmann area 7 [7m]), independently for children with obesity or who are overweight (*n* = 2,356; red) and children in a healthy weight range (*n* = 4,754; green). Higher BMI was associated to lower gFCD in precuneus, independently across weight categories, and to higher fALFF only in children with obesity or who are overweight. Right cerebral hemisphere. Black contours delineate the borders of 180 ROIs in the right cerebral hemisphere. BMI percentiles based on age and sex were used to determine weight categories.

To examine the associations between BMI, resting-state metrics (fALFF and gFCD), and cognitive composite scores as a function of family income, we measured their Pearson correlations within specific ROIs of a multimodal parcellation of the human cerebral cortex (27) across weight categories. Lower cognitive performance correlated with higher fALFF, predominantly in insula, cingulate, and somatomotor cortices and in subcortical and cerebellar regions (Figure 4A). This association was consistent across children of a healthy weight and children with obesity or who are overweight in both Discovery and Replication subsamples (Supplemental Figures 8 and 9; *R* > 0.14, *P* < 2 × 10^–8^). Moreover, lower family income was linked to higher fALFF in occipital and medial temporal areas, insula, mid cingulum, and the somatomotor cortex (Figure 4B), independently across weight categories in the Discovery and Replication subsamples (Supplemental Figures 8 and 9; *R* > 0.09, *P* < 0.001). Additionally, lower cognitive performance was associated with lower gFCD in DMN and frontoparietal network (FPN) regions alongside higher gFCD in lateral visual areas, the paracentral lobe, and mid cingulate cortex (Figure 4A); this relationship was consistent across weight categories and subsamples (Supplemental Figure 9; *R* > 0.74, *P* < 0.003). Furthermore, lower family income was correlated with lower gFCD in FPN and DMN regions and with higher gFCD in lateral visual areas, somatomotor cortex, paracentral lobe, and mid cingulate cortex (Supplemental Figures 9 and 10; *R* > 0.072, *P* < 0.007). The correlation patterns for fALFF and gFCD were largely complementary of one another, both in Discovery and Replication subsamples, such that brain regions with high correlation for fALFF had low correlation for gFCD (family income: *R*[379] > 0.64; total composite: *R*(379) > 0.52; *P* < 1 × 10^–20^). While the correlations reported are significant (ranging from approximately 0.06 to 0.15) and reproducible, they exhibit modest effect sizes, which were detectable due to the large sample size of the ABCD Study.

Higher average FA throughout the brain’s WM tracts was associated with elevated gFCD and lower fALFF, independently across weight categories (*R* > 0.14; *P* < 1.5 × 10^–8^; Figure 4C).

### Effect of BMI within subcortical regions and cerebellum.

We also evaluated associations between BMI, resting-state activity, and connectivity within subcortical ROIs. The positive associations of BMI with fALFF were reproducible bilaterally, in all 19 subcortical ROIs (*P* < 0.05, Bonferroni corrected) and did not differ between weight categories (Supplemental Figure 11); those with gFCD were reproducible only in the bilateral cerebellum. Within the cerebellum, there were reproducible positive associations with BMI for fALFF bilaterally in lobules IV, V, VIIIb, and IX that did not differ between weight categories. The slopes of the linear associations between BMI and gFCD were negative in several regions: bilaterally in lobules IV, VI, VIIb, VIIIa, and IX, as well as Crus I (excluding vermis), and in Crus II, V (right), and VIIIb (vermis). Additionally, these associations in the bilateral posterior cerebellum (lobules VIIb and VIIIa and vermis VIIIa) were weaker for children with obesity or who are overweight compared with children in the healthy weight range (*P* < 0.001, 2-tailed *t* test). 

### CMA.

**Figure 5:**
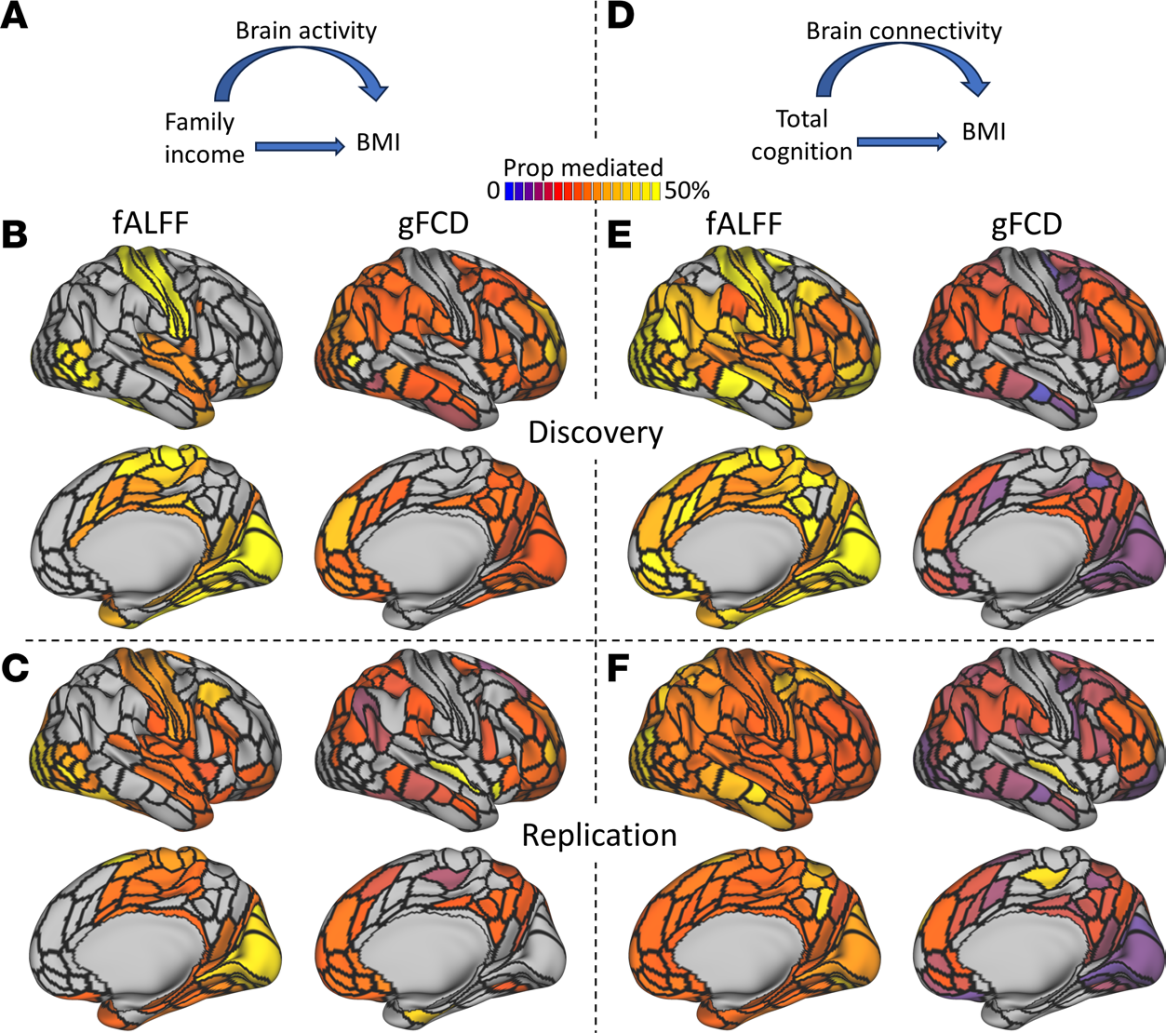
Causal mediation analysis (CMA; Model 1). Proportion of the total effects of family income (**A**–**C**) or cognitive performance (**D**–**F**) on the association with body mass index (BMI) that is mediated by the fractional amplitude of low-frequency fluctuations (fALFF), or global functional connectivity density (gFCD) overlaid on lateral and medial surfaces of the right cerebral hemisphere for the Discovery (*n* = 3,597 children) and the Replication sample (*n* = 3,513 children). Black contours delineate the borders of 180 ROIs in the right cerebral hemisphere. Threshold *P*_ACME_ < 0.001.

We investigated the indirect pathways linking family income and cognitive performance to BMI through fALFF or gFCD (CMA model 1; Figure 5, A and D). Our analysis revealed consistent indirect associations of family income on BMI, mediated through fALFF in bilateral somatomotor areas, insula, cingulum, and cerebellum, as well as through gFCD bilaterally in FPN and DMN regions (*P*_ACME_ < 0.001; Figure 5, B and C, and Supplemental Figures 12 and 13). Specifically, the bilateral superior frontal language (SFL), visual cortex (V3), and premotor (6d) areas demonstrated the strongest mediation effects of fALFF on the association between family income and BMI (>78%; Supplemental Figure 13). For family income, the average mediation proportion was higher for fALFF (Discovery, 37%; Replication, 36%) than for gFCD (Discovery, 14%; Replication, 7%; *t*[436] > 10; *P* < 2 × 10^–21^). Moreover, both fALFF and gFCD in these regions consistently acted as partial mediators for the association between cognitive performance and BMI (*P*_ACME_ < 0.001; Figure 5, E and F, and Supplemental Figure 14). For total composite scores, the average mediation proportion was also higher for fALFF (Discovery, 31%; Replication, 32%) than for gFCD (Discovery, 7%; Replication, 11%; *t*(624) > 14; *P* < 4 × 10^–42^). The regions where fALFF mediated the indirect effects of total cognition on the BMI association were more widespread than those observed for mediating the indirect effects of family income on BMI.

Additionally, we complemented our analysis by examining the indirect pathways linking the association of brain functional connectivity to BMI through family income and through cognitive performance (CMA model 2; Supplemental Figure 15). Our analysis revealed weak yet consistent mediation effects of family income on the associations between BMI and fALFF in bilateral insula and mid cingulum (Supplemental Figure 15) and between BMI and gFCD bilaterally in FPN and DMN regions (*P*_ACME_ < 0.001; Supplemental Figure 16). For family income, the average mediation proportion was higher for gFCD (Discovery, 5%; Replication, 4%) than for fALFF (Discovery, 2%; Replication, 2%; *t*(243) > 6.8; *P* < 1 × 10^–10^). Additionally, cognitive performance consistently acted as a modest mediator between fALFF or gFCD in these regions and BMI (*P*_ACME_ < 0.001; Supplemental Figures 15 and 16). Specifically, for total composite scores, the average mediation proportion was higher for gFCD (Discovery, 9%; Replication, 9%) than for fALFF (Discovery, 7%; Replication, 6%; *t*(293) > 3.9; *P* < 1 × 10^–4^). The proportion of mediation from CMA model 1 was greater than that from CMA model 2.

## Discussion

The high prevalence of obesity in American children has raised concerns about its implication to brain development, as evidence is emerging that obesity in children is linked to adverse effects on brain function and structure and on cognition (5–10). Access to the large longitudinal data set from the ABCD has increased the power to investigate the effects of obesity on brain structure (including WM integrity), function (including functional connectivity), and cognition (28, 29). However, the reproducibility and implications of these effects (30) remain unclear. Here we investigated the effects of BMI on DTI and resting-state functional MRI (fMRI) metrics using the large cohort of U.S. children from the ABCD Study while strictly controlling for confounding demographic variables (e.g., age, sex, race), head motion, and brain volume, and study-specific variables (scanner manufacturer and research site) separately in Discovery and Replication samples. We found that BMI was associated positively with spontaneous brain activity, as indexed by fALFF, and negatively with brain connectivity (structural and functional), family income, and cognition, reproducibly in the Discovery and Replications subsamples. The spontaneous brain activity (predominantly in somatomotor areas) partially mediated the outcomes, such that close to 80% of the total effects of family income and cognitive performance on their association with BMI were mediated by fALFF in the somatomotor cortex. This suggests that changes in socioeconomic status or in cognitive performance may influence BMI partly through their effect on brain activity in these brain regions. Notably, the mediation effects of fALFF on the association between cognitive performance and BMI were more widespread in the brain than those observed for family income, indicating that cognition contributes to BMI independently of family income.

We found a reproducible association between elevated BMI and reduced lD in the corpus callosum (Cohen’s d = 0.49), along with a less pronounced yet still significant association with other WM tracts. Axial diffusivity (lD) measures the rate of diffusion of water molecules along the primary axis of WM fibers and provides information about WM microstructural integrity (31). The corpus callosum plays a crucial role in functional lateralization and in the coordination of cognitive, sensory, and motor systems that are needed for conscious experience (32). Our findings are consistent with those of prior DTI studies in adults and adolescents that reported correlations between BMI and decreased FA or increased MD in the corpus callosum (28, 33–41), and they are consistent with the notion that WM integrity is compromised in obesity (29) (see review by Kullmann et al.; ref. 8). The lower longitudinal water diffusion in this region may indicate reduced interhemispheric connectivity, which could reduce the integration of information between left and right cortical areas and contribute to the cognitive impairment reported in children with high BMI (42, 43). The reproducible linear associations of FA with fALFF and gFCD are consistent with the assumption that brain activity and functional connectivity are influenced by the structural connectivity of the brain (44).

Higher BMI was associated with increased fALFF in interoceptive, somatomotor, medial visual, subcortical, and cerebellar regions (Cohen’s d = 0.11). This suggests increased local neuronal activity in these regions in children with obesity or who are overweight compared with children in a healthy weight range. These findings are consistent with reports of higher synchrony or amplitude of spontaneous resting activity in the brain for men who are obese compared with men who are healthy weight (45, 46).

In contrast to the positive association between BMI and fALFF, the association with gFCD was negative and predominantly affected multimodal association cortices. Children with high BMI displayed lower gFCD, with the strongest effects in default-mode and cingulo-opercular regions (Cohen’s d = 0.16). gFCD maps the overall functional integration of brain regions (21), contrasting the few metabolically demanding hubs (47) that orchestrate major resting state networks (48) with the abundant weakly interconnected brain network nodes (49). The strongest association with gFCD was in the precuneus, which is one of the main hubs in the brain (49) that engages in highly integrated internally and externally driven processes (50). The opposite pattern between fALFF and gFCD with BMI is reminiscent of a pattern we previously reported for the effects of methylphenidate (51). Though methylphenidate is prescribed to improve attention in ADHD children (52), it also leads to weight loss and has been used to reduce weight in children with obesity (53). Increased gFCD in precuneus was associated with higher cognitive scores in the current study, suggesting that reduced gFCD might contribute to processes that increase risk for obesity and impair cognitive performance.

Our findings were reproducible in Discovery and Replication subsamples and align with prior findings of DMN hypoconnectivity in individuals with obesity or who are overweight (54). These findings suggest that obesity is associated with perturbations of brain network connections involved in self-referential processing. Crucially, BMI-related decreases in both gFCD within the DMN and lD in corpus callosum were positively correlated, both in Discovery and Replication subsamples, indicating that BMI affects both functional and structural brain connectivity. This finding aligns with the role of the corpus callosum in facilitating functional connectivity across distributed networks (55). Since the ABCD Study is longitudinal, monitoring these children over time will enable the assessment of whether a high BMI triggers disruptions in both structural and functional connectivity. It will also help determine whether improvements in connectivity are evident in children who lose weight but not in those who do not. Additionally, examining whether disrupted connectivity in nonoverweight or children without obesity can predict the future development of obesity would suggest that impaired connectivity might serve as a vulnerability factor, increasing the risk for obesity.

In the present study, the fluid and total cognitive composites were lower for children with obesity than children in a healthy weight range and decreased in proportion to BMI in children with obesity, consistent with the negative association between BMI and executive function in ABCD children (7). Children exhibiting lower cognitive composite scores in our study also demonstrated reduced lD in the corpus callosum and lower gFCD in the precuneus. These findings align with our hypothesis that impairments in fluid cognition reflect lower information integration in DMN regions (22) and may be influenced by BMI-related factors. Note that the lack of associations between the crystalized composite score and BMI is consistent with prior ABCD studies that reported BMI-related decreases for total and fluid cognition but not for crystallized cognition (56). Though the mechanisms associated with reduced lD and lower gFCD in children with obesity are unclear, they might reflect in part obesity-related neuroinflammatory changes (57).

Higher family income was associated with lower BMI, consistent with the negative relationship between household income and BMI in U.S. children that reflects in part the lower costs of obesigenic than healthy foods (58). Income was also positively associated with lD in corpus callosum and with gFCD in DMN regions, consistent with our prior findings (15). Various studies have shown that children from lower-income families had worse cognitive performance (59), thinner cortices and smaller cortical surface area and volume (14, 60–65), lower brain activation during working memory (66) and decision-making (67) fMRI tasks, lower FA (68), and greater tendency to become obese or overweight in adolescence (69). These data suggest that excess body weight, which likely reflects multiple factors (improper diet, reduced physical activity, impaired metabolism, genetics, environmental toxins, endocrinological conditions, insufficient sleep, stress, and hormonal imbalances as well as preexisting executive control dysfunction), contributed to the reduced WM diffusion in corpus callosum and DMN functional connectivity we observed in the children from low-income families.

Here we document partial mediation effects of fALFF in the relationships between both family income and cognitive performance with BMI. Common brain regions that mediated the association between fALFF and obesity for income and cognition included the somatomotor cortex, insula, cingulum, and cerebellum. The identification of insula and the cingulum, regions of the cingulo-opercular network (CON) that is involved in executive control (70), as mediators between both family income and cognitive performance with BMI is in line with the role of the salience network, which is part of the CON and plays a role in the control of impulsive behavior to high calorie food stimuli in children (71–73). Since greater impulsivity and a preference for high-calorie foods has been observed in studies of preschoolers with obesity or are are overweight (74), our CMA findings suggest that higher spontaneous brain activity within regions such as the insula and cingulum may contribute to differences in BMI, potentially through their influence on impulsive behaviors related to food consumption. Furthermore, the mediation of fALFF in somatomotor and cerebellar regions is consistent with their role in motor control, sensory processing, reward integration, impulse regulation, and coordination during eating (75–77).

Though these findings indicate that socioeconomic factors influence BMI partly through their impact on brain activity (fALFF), it is noteworthy that the mediation of fALFF in the indirect effects of cognitive performance on BMI showed a more widespread pattern in the brain than that observed for family income. This indicates that the contribution of regional brain activity to the indirect effects of cognition on the association to BMI goes beyond that mediated through family income. This likely reflects the fact that multiple factors contribute to cognition in children beyond family income, including the quality of education, richness of exposures, nutrition, sleep, physical activity, and genetics among others (78). Our findings are relevant to public health since they indicate that interventions and policies that provide support to low-income family would improve cognitive performance and brain development, as recently shown by a study based on ABCD (79). Our findings also suggest that prevention interventions that support parents on how to improve cognitive skills in children (80) could be beneficial to brain development and reduce the risk for obesity. It also suggests that strengthening the educational system might help prevent obesity in children.

Our findings reveal a negative correlation between BMI and family income, even among healthy weight individuals. This suggests that the association between BMI and socioeconomic status is not limited to obesity but extends across the entire range of BMI values. Several factors may contribute to this broader relationship. Higher-income families are likely to have better access to healthier food options, opportunities for physical activity, and healthcare resources, all of which support maintaining a healthy weight (81). Additionally, higher educational attainment associated with higher income levels may lead to better knowledge and practices regarding nutrition and health (82). Environmental factors also play a role, as higher-income families often live in neighborhoods with more recreational facilities and safer environments for physical activity (82). Moreover, lower-income families may face higher levels of stress and mental health challenges (83), contributing to weight gain and higher BMI through stress-related eating behaviors and reduced opportunities for physical activity (84). These findings underscore the importance of considering socioeconomic factors in the study of BMI and weight-related health outcomes. Interventions aimed at reducing obesity and improving overall health should consider the broader socioeconomic context and address the disparities in resources, education, and environmental factors that influence BMI.

For CMA model 1, the average mediation proportion was higher for fALFF compared with gFCD. This suggests that spontaneous brain activity may have a stronger influence on the relationship between family income and cognitive performance with BMI compared with functional connectivity. While both measures reflect different aspects of brain function, this difference in mediation proportions could reflect the specific roles these brain processes play in the regulation of eating behaviors and metabolic processes. CMA model 1 showed greater mediation proportions compared with CMA model 2, suggesting that family income and cognitive performance influence brain activity and connectivity directly, thus increasing their associations with BMI. Our CMA findings also highlight the complex interplay between socioeconomic factors, brain function, and BMI during childhood. While our CMA suggests a pathway in which impaired cognition influences BMI (CMA model 1), it is important to recognize that the relationship is likely to be bidirectional, with obesity-related metabolic consequences potentially affecting cognitive function (CMA model 2). Furthermore, both BMI and cognitive function could be influenced by other factors, such as socioeconomic status, lifestyle choices, or genetic predispositions. Therefore, our findings should not be interpreted as to conclude that high BMI in youth is solely due to cognitive deficits but instead as part of a complex interplay of multiple factors influencing both cognition and BMI. The direction of causality in Figure 5D might seem counterintuitive. However, in our analysis, we used total cognition scores as a proxy for cognitive stimulation. Cognitive stimulation, which encompasses various activities that challenge and engage the brain, can play a crucial role in shaping and enhancing brain connectivity, particularly during critical developmental periods. By representing cognitive scores as influencing connectivity, we aim to highlight the dynamic and reciprocal nature of this relationship. Cognitive stimulation, reflected in higher cognitive performance scores, can lead to improvements in brain connectivity, just as robust connectivity can support better cognitive function. This bidirectional relationship underscores the importance of considering both directions of influence in understanding brain-behavior interactions.

Other limitations of our study include the restricted age range of participants, which may limit the applicability of findings to other stages of brain development. Moreover, the underrepresentation of very-low-income families in the ABCD Study compared with the broader U.S. population should be noted. While parental education levels align at lower tiers between the ABCD sample and the U.S. population, a relatively higher percentage of parents in the ABCD Study attained a bachelor’s degree compared with the U.S. population. The magnitudes of most effects in this study are quite modest, and they achieve statistical significance primarily due to the very large sample size of the ABCD data set. BMI was also negatively correlated with family income even among healthy weight individuals, whose BMI typically ranged from 15 to 20. This suggests that the association between BMI and family income is not solely driven by obesity but reflects broader socioeconomic influences that affect individuals across the entire BMI spectrum.

In summary, we demonstrate consistent, modest associations between BMI and cognitive performance, family income, spontaneous brain activity, and functional and structural brain connectivity in 9- to 10-year-old children. The association between poor cognitive performance and BMI partially reflects increased spontaneous brain activity in the salience network and somatomotor and cerebellar regions that is accentuated in children from low-income households. Although our data suggest that low income and impaired cognition influence BMI in part thorugh their effects in brain, it is also likley that these associations are bidirectional. High BMI, with its adverse metabolic effects such as neuroinflammation, likely affects both the brain and cognition.

## Methods

Supplemental Methods are available online with this article.

### Sex as a biological variable.

Findings from this study do not apply exclusively to 1 sex, as both girls and boys were included in the analysis. Specifically, both girls (*n* = 3,414) and boys (*n* = 3,696) participated in the study, ensuring representation from both sexes. Sex was defined at birth and was determined based on biological characteristics. The ABCD Study, from which the data for this study were derived, collected both sex and gender data, ensuring comprehensive data collection practices. There were no significant sex differences in the effect of BMI on brain connectivity. Consequently, sex was considered as a covariate of no interest in the statistical analysis to account for any potential variability related to sex.

### Participants.

The multisite longitudinal ABCD Study follows over 11,800 children into early adulthood for 10 years with annual lab-based assessments and biennial MRI. Children were excluded if they had medical, neurological, or cognitive problems; poor English-language proficiency; or contraindications for MRI (85).

In the present study, we analyzed baseline neuroimaging and behavioral data from 9,521 children in the ABCD Study reported in the 2.0 data release for whom WM diffusion metrics and resting-state fMRI data in the Connectivity Informatics Technology Initiative (CIFTI) format were available. In the analysis of functional connectivity, we excluded 560 participants with excessive levels of head motion during resting-state fMRI (>50% of time points with framewise displacement, FD < 0.5mm), 282 underweight (BMI < fifth percentile), and 284 participants missing critical information (BMI, cognitive composite scores, or family income). We restricted the study to African American, Hispanic, and White groups to minimize variability in our analysis. This approach helps ensure a more homogenous sample and reduces potential confounding factors related to ethnicity. Consequently, we excluded 1,105 participants of Asian (*n* = 162) or mixed (*n* = 943) race/ethnicity to maintain this focus and enhance the reliability of our findings within the specified groups. Thus, the final sample for studies on BMI and resting-state functional connectivity included 7,290 children (3,501 girls and 3,789 boys). The study on structural connectivity metrics was restricted to 4,797 of these participants (2,386 for Discovery and 2,411 for Replication; 2,283 girls and 2,514 boys) who underwent MRI on Siemens scanners to minimize the variability of DTI metrics across MRI scanners in the ABCD Study (86).

### BMI.

The children’s BMI was extracted from the ABCD Youth Anthropometrics data (abcd_ant01.txt), which was downloaded from the National Institute Mental Health Data Archive (NDA; https://nda.nih.gov/). We used the clinical growth charts provided by the National Center for Health Statistics at the Center for Disease Control and Prevention (CDC) to determine BMI percentiles based on age and sex (https://www.cdc.gov/growthcharts/clinical_charts.htm) to determine categories for healthy weight (fifth percentile < BMI < 85th percentile) and overweight/obese (BMI > 85th percentile).

### Behavioral data.

We downloaded standard fluid, crystallized, and total cognition composite scores from NDA, which were calculated within the NIH Toolbox (23). The uncorrected fluid composite scores were calculated using the following tests: (a) pattern comparison processing speed; (b) list-sorting working memory;(c) picture sequence memory; (d) Eriksen flanker task; and (e) the dimensional change card sort task. The crystallized composite scores were calculated using (f) the oral reading recognition and (g) the picture vocabulary tests. The fluid and crystallized composites were used to calculate the total cognition composite scores.

### Family income.

The ABCD Study surveyed the annual household income using 10 income brackets: (a) < $5,000; (b) $5,000–$12,000; (c) $12,000–$16,000; (d) $16,000–$25,000; (e) $25,000–$35,000; (f) $35,000–$50,000; (g) $50,000–$75,000; (h) $75,000–$100,000; (i) $100,000–$200,000; (j) > $200,000. These data were downloaded from NDA.

### Depression.

To assess impairments in functioning due to depression, we used the ABCD Parent Diagnostic Interview (abcd_ksad01), which was downloaded from NDA.

### MRI data.

For functional connectivity analyses, we used the ABCD–brain imaging data structure (ABCD-BIDS) Community Collection (ABCC) (https://collection3165.readthedocs.io/en/stable/), which includes resting-state fMRI data from 10,038 children that have passed quality assurance (87). ABCD-BIDS used a modified version of the Human Connectome Project (HCP) pipeline to accommodate GE, Phillips, and Siemens scanners and head coils from all 21 ABCD sites, which minimizes unwanted variability from differences in MRI scanners. The ABCD imaging procedures were standardized for 3T MRI scanners (Siemens Prisma, Phillips, and General Electric 750 scanners) that were equipped with adult-sized multichannel coils and capable of performing multiband echo planar imaging (EPI). These procedures were implemented across 21 sites, and further details can be found in refs. 86 and 88. In summary, structural MRI employed 3D T1w inversion-prepared RF-spoiled gradient echo and T2w variable flip angle fast spin echo pulse sequences with 1 mm isotropic resolution. fMRI data were acquired using T2*-weighted multiband EPI with parameters including TE/TR of 30/800 ms, 2.4 mm isotropic resolution, a flip angle of 52°, 60 slices covering the entire brain, and a multiband slice acceleration of 6 (88). Diffusion MRI data with 1.7 mm isotropic resolution were acquired using multiband EPI (89, 90) with slice acceleration factor = 3, five b-values (b = 0, 500, 1000, 2000, and 3000 s/mm^2^), and 96 diffusion directions (86). In the ABCD 2.0 data release, a probabilistic method was employed to automatically label all major WM tracts (91) while excluding GM and cerebral spinal fluid (CSF) voxels (86).

### Reproducibility.

Participants were split into 3 independent demographically matched subsamples: Discovery (*n* = 3,597, girls = 1,765), Replication (*n* = 3,513, girls = 1,649), and Normality (*n* = 180; girls = 87) using ABCC’s “matched group” status, which is based on sociodemographic factors that can affect brain development (age, sex, ethnicity, grade, highest level of parental education, handedness) (87).

### Quality assurance.

The automated QA procedures of the ABCD Study are described elsewhere (86). Additionally, images underwent correction for scanner-specific gradient distortions and intensity irregularities. Trained evaluators reviewed the images for potential issues like low quality and artifacts such as blurriness, ghosting, or ringing, which might hinder brain segmentation (86).

### ABCD-BIDS pipeline.

Like the HCP pipeline, the ABCD-BIDS pipeline is composed of 5 consecutive steps: *PreFreesurfer*, performs brain extraction, denoising, and normalization of structural data to a standard template; *Freesurfer*, performs brain segmentation and creates cerebral surfaces with FreeSurfer (86), which has been validated for use in children (92); *PostFreesurfer*, converts brain surfaces into the HCP-compatible CIFTI format; *fMRIVolume*, registers the functional time series to the volumetric standard template; and *fMRISurface*, converts functional time series data to the CIFTI format. Differences between the HCP and ABCD-BIDS pipelines are fully described elsewhere (87). Briefly, the ABCD-BIDS pipeline does not require T2w images and performs the nonlinear registration to the standard atlas in *PostFreeSurfer*, which increases the effectiveness of the registration. Additionally, the ABCD-BIDS pipeline uses ANTS (93) for nonlinear registration, which consistently outperforms other nonlinear registration methods (94). In addition, the *fMRISurface* step in the ABCD-BIDS pipeline includes functional connectivity preprocessing that separates true head motion from fictitious motion induced by breathing-related magnetic field changes (95); it performs standard denoising by regressing out time-varying head motion, WM and CSF signals, and global signals that may affect group comparisons (96, 97). This process is applied to both dense (dtseries) and parcellated (ptseries) CIFTI data sets within the 360 cortical partitions (98) and the 19 subcortical partitions obtained from Freesurfer.

### Head motion.

Motion-censoring data, determined using the ABCD-BIDS pipeline, was utilized to eliminate time frames with FD > 0.5 mm. Addressing head motion is crucial in pediatric structural and functional neuroimaging (99). To address this, we also considered individuals’ average FD during resting-state fMRI scans as an indicator of their head movement tendencies while in the scanner.

### Structural connectivity.

To assess WM integrity from diffusion tension imaging measures of FA, rD, lD, and MD, we used tabulated diffusion imaging metrics, which were downloaded from NDA and are described elsewhere (86).

### fALFF and gFCD.

The fALFF was used to quantify the proportion of resting fMRI signal fluctuations in 0.01–0.10 Hz low-frequency bandwidth (20), a marker of brain activity (100). gFCD mapping (49) was used to quantify the density of functional connections at a given brain coordinate with all other brain coordinates. gFCD was equated to the logarithm of the total number of functional connections, which was computed using Pearson correlation (49). Specifically, 2 grayordinates were considered functionally connected if their time-varying signals had a correlation *R* > 0.6 (21). fALFF and gFCD were mapped at each brain grayordinate (47) from individual time series with *n* = 91,282 grayordinates (101) and a maximum of 1,520 time points (20 minutes) using Matlab 2017b (MathWorks) and the Biowulf cluster at NIH (https://hpc.nih.gov/).

### ROI analysis.

Average ROI values within each of the 379 partitions and 28 cerebellar partitions (102) were independently computed for each individual to assess the associations of fMRI metrics (fALFF and gFCD) with cognition and family income.

### Functional specialization index.

To assess the overall functional specialization of the ROIs, we used the multimodal parcellation of the human cerebral cortex (98), which documents the degree of associations with 3 auditory, somatomotor, and visual domains for each ROI. Specifically, the functional specialization index was defined in terms of the absolute differences in specialization between domains S_1_ = auditory versus somatosensory; S_2_ = auditory versus visual; and S_3_ = somatosensory versus visual as functional specialization index = max(S_i_) – mean(S_i_) and was normalized to 1 across 360 atlas partitions (26).

### Mediation analysis.

The “mediation” package (103) was used to estimate causal mediation effects (104). One thousand bootstrapping samples and a heteroskedasticity-consistent estimator for the covariance matrix were used to estimate the average direct effects (ADE) and average causal mediation effects (ACME) and the mediated proportion.

### Statistics.

In the independent Normality subsample, we confirmed the normal distribution of imaging metrics using the Shapiro-Wilk normality test (105) (W > 0.98; *P* > 0.05). A 2-tailed *t* test was used to assess group differences in family income and cognitive composite scores Before statistical analysis, we removed site- and scanner-specific differences using grand mean scaling, regressed out effects of head motion and brain volume across participants independently for boys and girls, and removed effects associated with race. Then, a factorial ANCOVA was conducted in MATLAB, independently for the Discovery and Replication subsamples, to assess the main effects of BMI on the dependent variable Y (fALFF or gFCD) using a sex covariate. In follow-up ROI analyses, the effects of BMI and sex on Y (FA, MD, lD, rD, fALFF, or gFCD) were assessed using ANCOVA in R. We used a FDR threshold pFDR < 0.05 to correct for multiple comparisons across 91,282 grayordinates or 379 ROIs; for the DTI measures, we used Bonferroni corrections across 42 major WM bundles in the AtlasTrack (91). Pearson correlation analysis was conducted in R to assess the associations of average brain metrics (Y) within specific ROIs with cognitive composite scores and family income.

### Study approval.

Local IRB at 21 data collection sites across the United States and the IRB at the UCSD approved the ABCD Study (106). Recruitment replicated demographic characteristics of the general U.S. population (107). Children provided written assent for their participation, and parents provided written informed consent.

### Data availability.

ABCD data are publicly available through the National Institute of Mental Health Data Archive (https://data-archive.nimh.nih.gov/abcd). Values for all data points in graphs are reported in the Supporting Data Values file.

## Author contributions

DT and NDV designed the research, DT analyzed data, and DT and NDV wrote the manuscript.

## Supplementary Material

Supplemental data

ICMJE disclosure forms

Supporting data values

## Figures and Tables

**Table 1:**
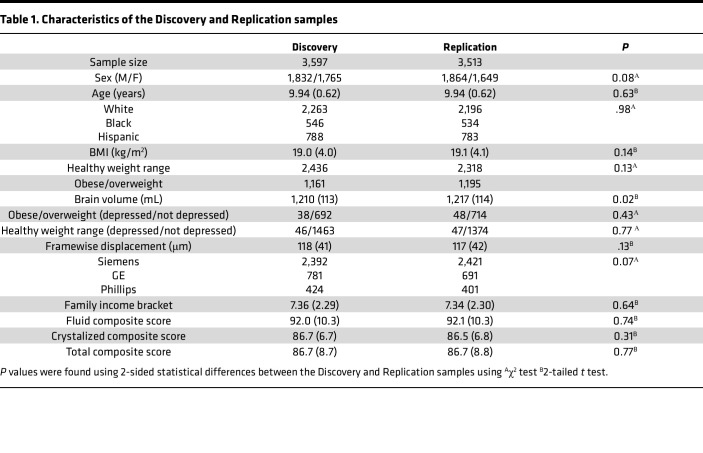
Characteristics of the Discovery and Replication samples

## Version 1 07/09/2024

In-Press Preview

## Version 2 08/22/2024

Electronic publication
