# Supplementary material for: Childhood obesity’s effect on cognition and brain connectivity worsens with low family income
Source: JCI Insight. 2024 Jul 9;9(16):e181690. doi: 10.1172/jci.insight.181690 (PMC11343596; doi:10.1172/jci.insight.181690)
Supplement: Supplemental data [file jciinsight-9-181690-s106.pdf]

Supplemental Methods and Results for

Childhood Obesity's Impact on Cognition and Brain Connectivity Worsens with Low Family Income

Dardo Tomasi*[1] and Nora D. Volkow[1,2]

[1]National Institute on Alcohol Abuse and Alcoholism, Bethesda, MD, 20892

[2]National Institute on Drug Abuse, Bethesda, MD, 20892

Recruitment. Information regarding recruitment methods and criteria for the ABCD study can be found in published materials accessible on the ABCD website (https://abcdstudy.org/scientists/protocols/). In short, the ABCD study employed probability-based sampling of U.S. schools located within 21 catchment areas, which are geographical regions centered around schools within a 50-mile radius of the research institution, as the primary approach for identifying and enrolling eligible children and their parents (1). Families were given recruitment materials and digital copies. Families expressing interest underwent a short telephone screening. If eligible, they were enrolled and scheduled for the baseline assessment at the research centers. Both guardians and children received compensation for their participation (1). Recruitment closely represented demographic variables (sex, race, ethnicity, parental marital status and education, and income) of the general US population (2).

Inclusion and Exclusion criteria: Children were included unless they had severe psychiatric or neurological disorders or significant medical conditions. Exclusion criteria comprised common MRI contraindications, limited fluency in English, uncorrected sensory impairments, major neurological disorders in their medical history, very premature birth (<28 weeks), extremely low birth weight (<1,200 g), prolonged hospitalization due to birth complications, current diagnosis of schizophrenia, moderate to severe autism spectrum disorder, a history of traumatic brain injury, or unwillingness to participate in assessments (3, 4). Children, whether taking medications or not, were considered for inclusion.

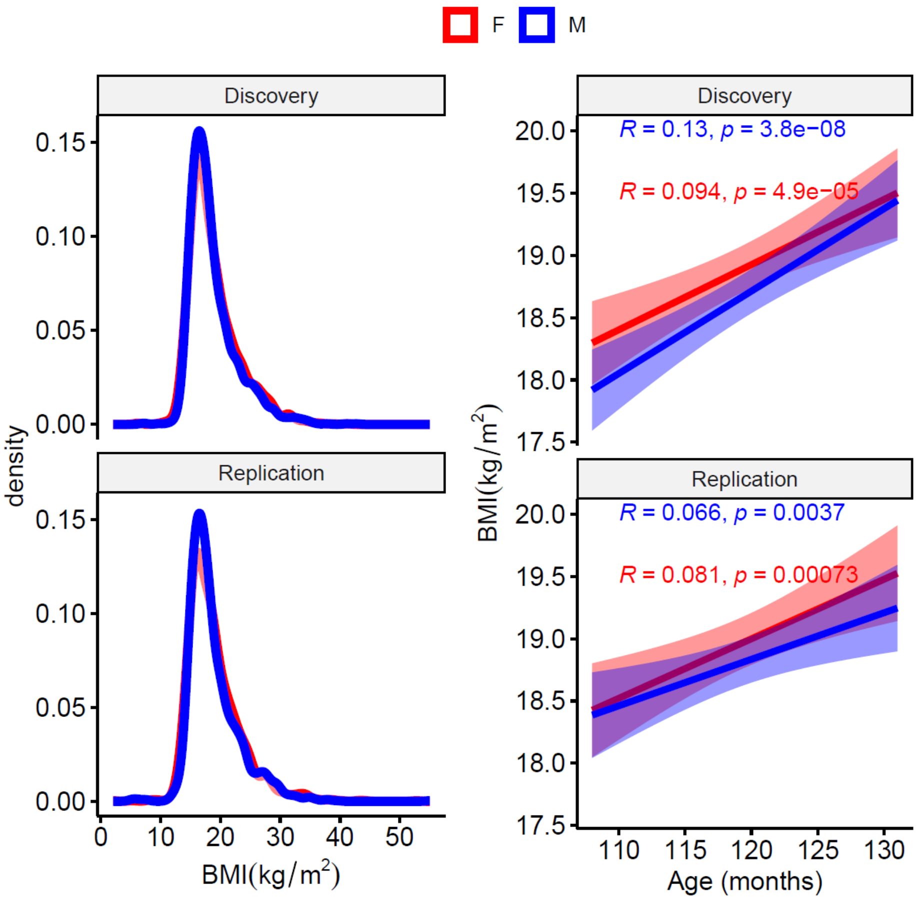

Supplemental Figure S1: Reproducibility of BMI characteristics by sex. Distribution of BMI (left) and its age-related increases (right) among 3,696 boys and 3,414 girls, and their reproducibility in Discovery (n=3,597) and Replication (n=3,513) subsamples.

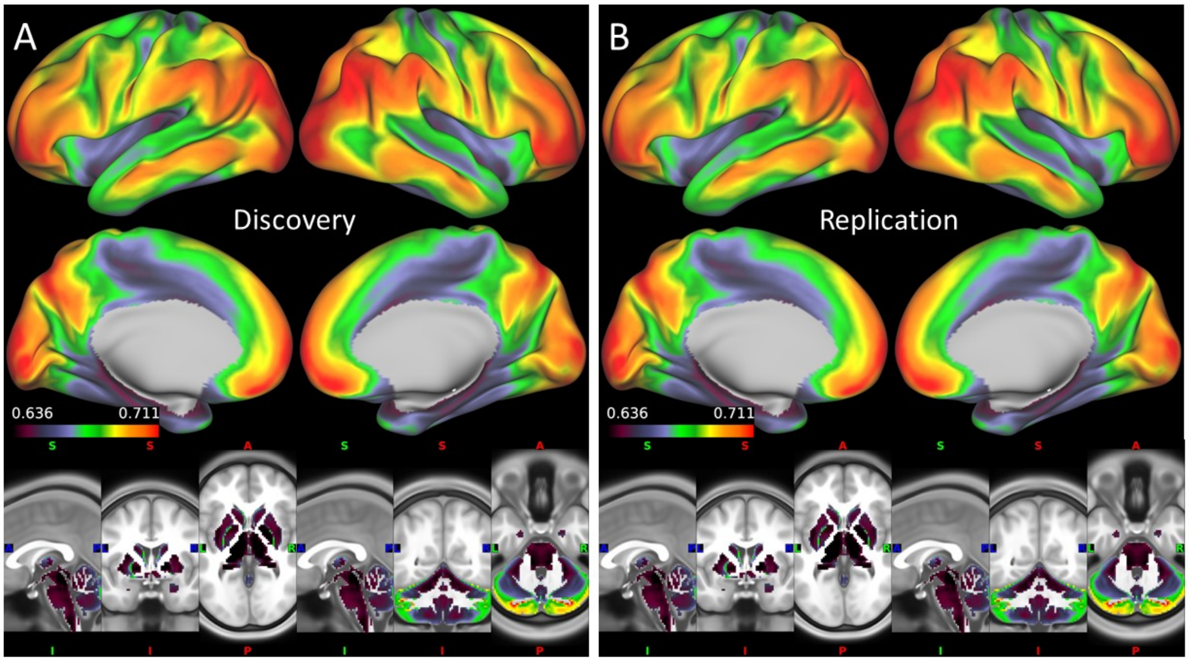

Supplemental Figure S2: Reproducibility of fALFF patterns. Average fractional amplitude of low-frequency fluctuations (fALFF) overlaid on lateral and medial cerebral surfaces and 6 orthogonal views of a template brain for the *Discovery* (**A**; n=3,597 children) and the *Replication* sample (**B**; n=3,513 children).

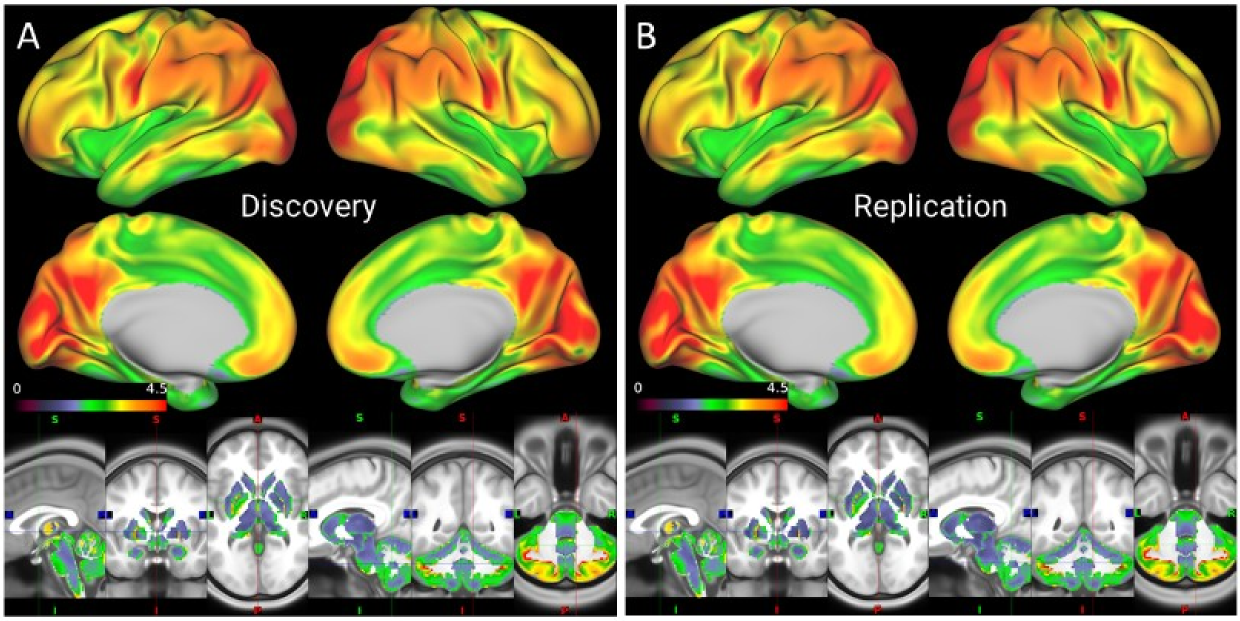

Supplemental Figure S3: Reproducibility of gFCD patterns. Average global functional connectivity density (gFCD) overlaid on lateral and medial cerebral surfaces and 6 orthogonal views of a template brain for the *Discovery* (**A**; 3,597 children) and the *Replication* sample (**B**; 3,513 children).

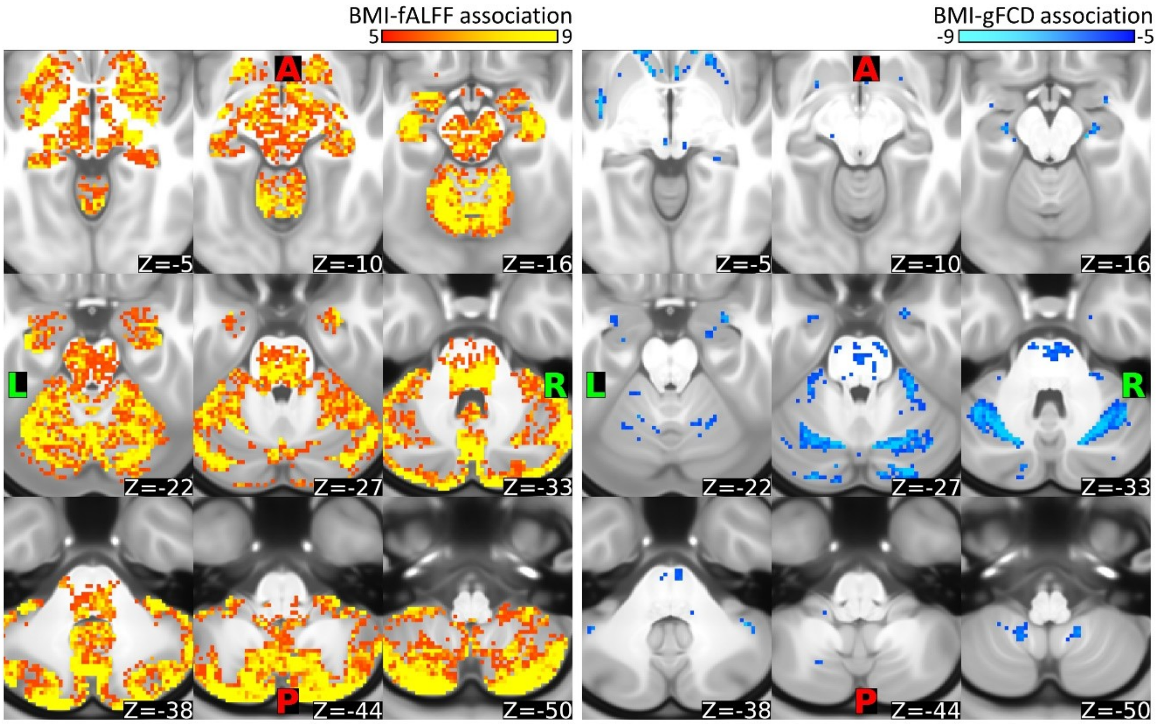

Supplemental Figure S4: BMI-associations in subcortical. Statistical significance (t-score) for the effect of body mass index (BMI) on the fractional amplitude of low-frequency fluctuations (fALFF; left) and global functional connectivity density (gFCD; right) across 7,110 children overlaid 9 axial views depicting subcortical and cerebellar brain regions. Statistical model: ANCOVA.

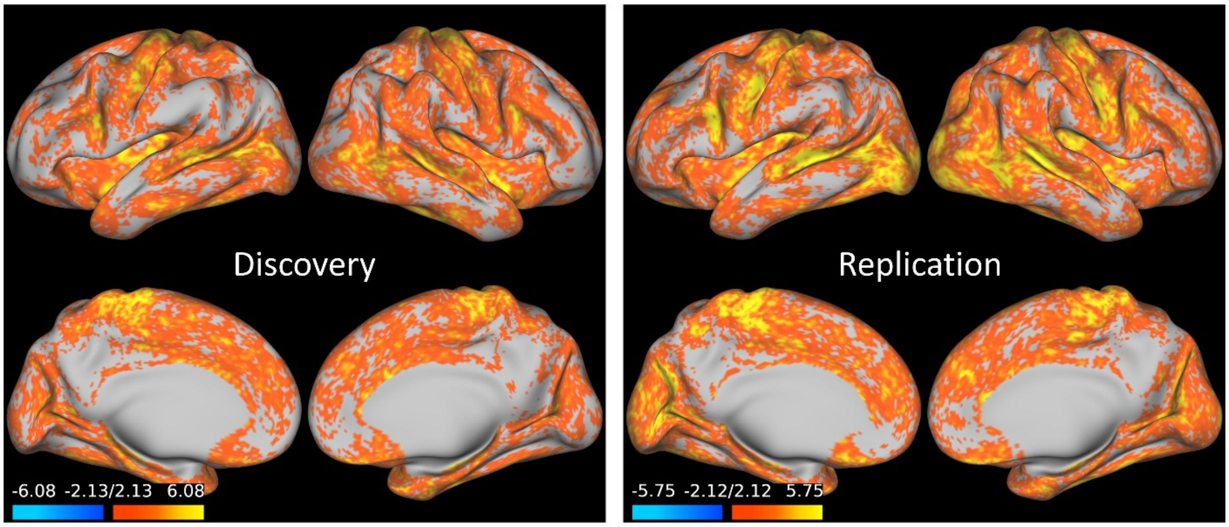

Supplemental Figure S5: Reproducibility of BMI-fALFF associations. Statistical significance (t-score) for the effect of body mass index (BMI) on the fractional amplitude of low-frequency fluctuations (fALFF) overlaid on lateral and medial cerebral surfaces for the *Discovery* (n=3,597 children) and the *Replication* sample (n=3,513 children). An FDR-corrected threshold P<0.05 was used to control for multiple comparisons across 91,282 grayordinates. Statistical model: ANCOVA.

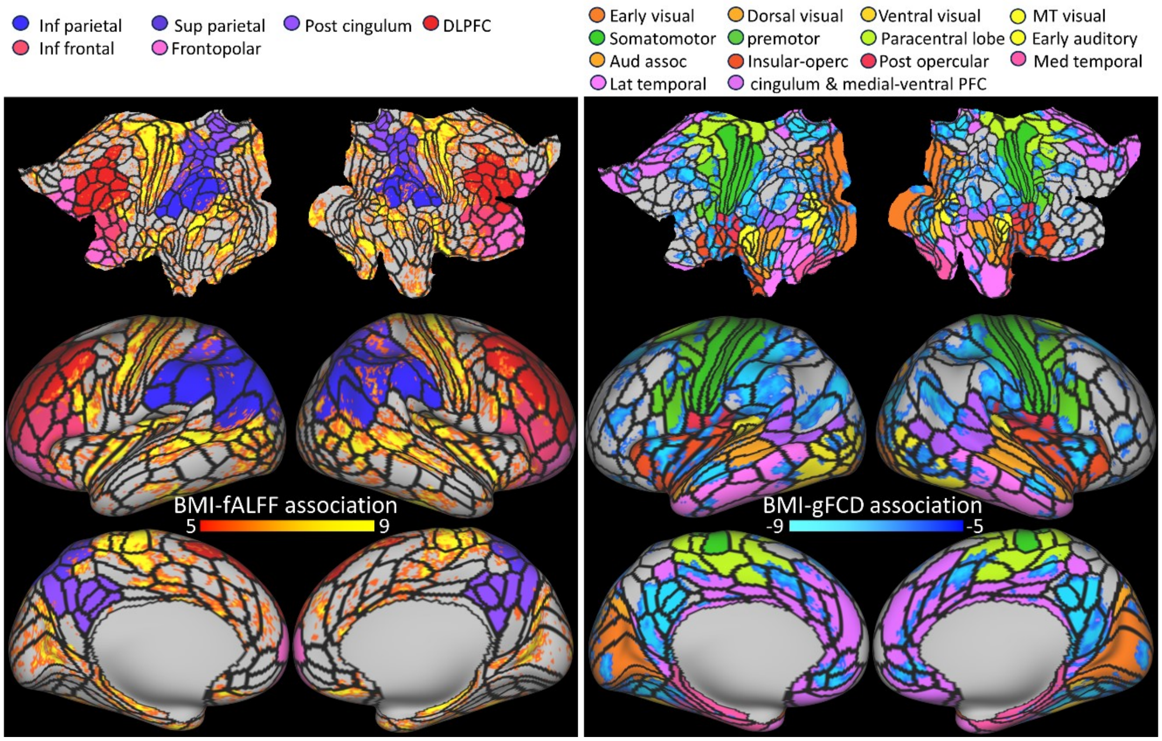

Supplemental Figure S6. Effect of BMI on fALFF and gFCD. Statistical significance (t-score) for the effect of body mass index (BMI) on the fractional amplitude of low-frequency fluctuations (fALFF; **a**) and global functional connectivity density (gFCD; **b**) across 7110 children, and the score of a functional specialization index (see text) rendered on flat (top row) and lateral and medial inflated surfaces (middle and bottom rows) of the left (L) and right (R) cerebral hemispheres. Black lines are the contours of 360 multi-modal partitions of the human cerebral cortex.(5) Statistical model: ANCOVA.

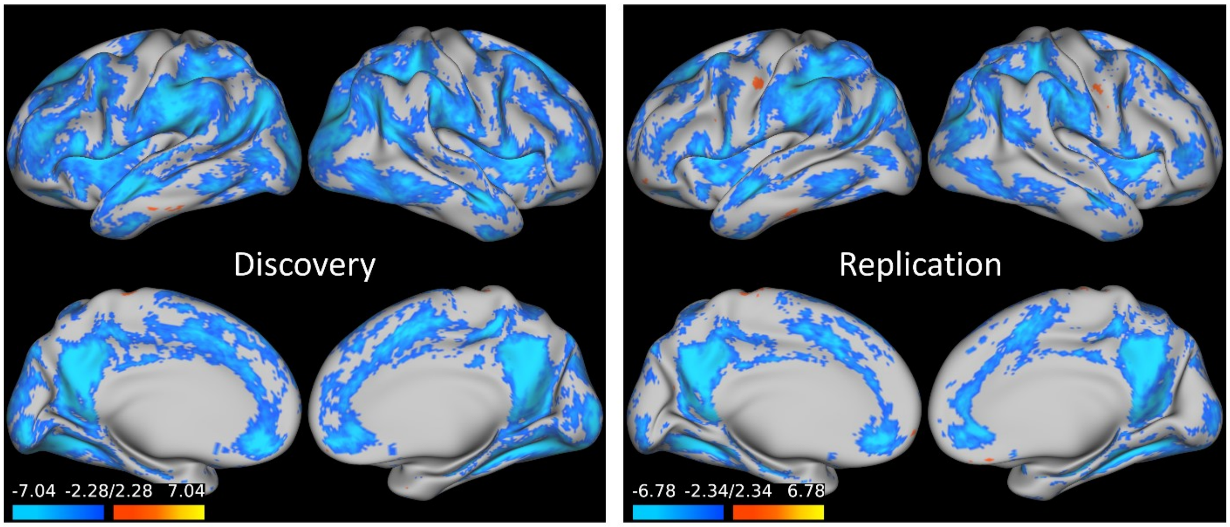

Supplemental Figure S7: Reproducibility of BMI-gFCD associations. Statistical significance (t-score) for the effect of body mass index (BMI) on the global functional connectivity density (gFCD) overlaid on lateral and medial cerebral surfaces for the *Discovery* (n=3,597 children) and the *Replication* sample (n=3,513 children). An FDR-corrected threshold P<0.05 was used to control for multiple comparisons across 91,282 grayordinates. Statistical model: ANCOVA.

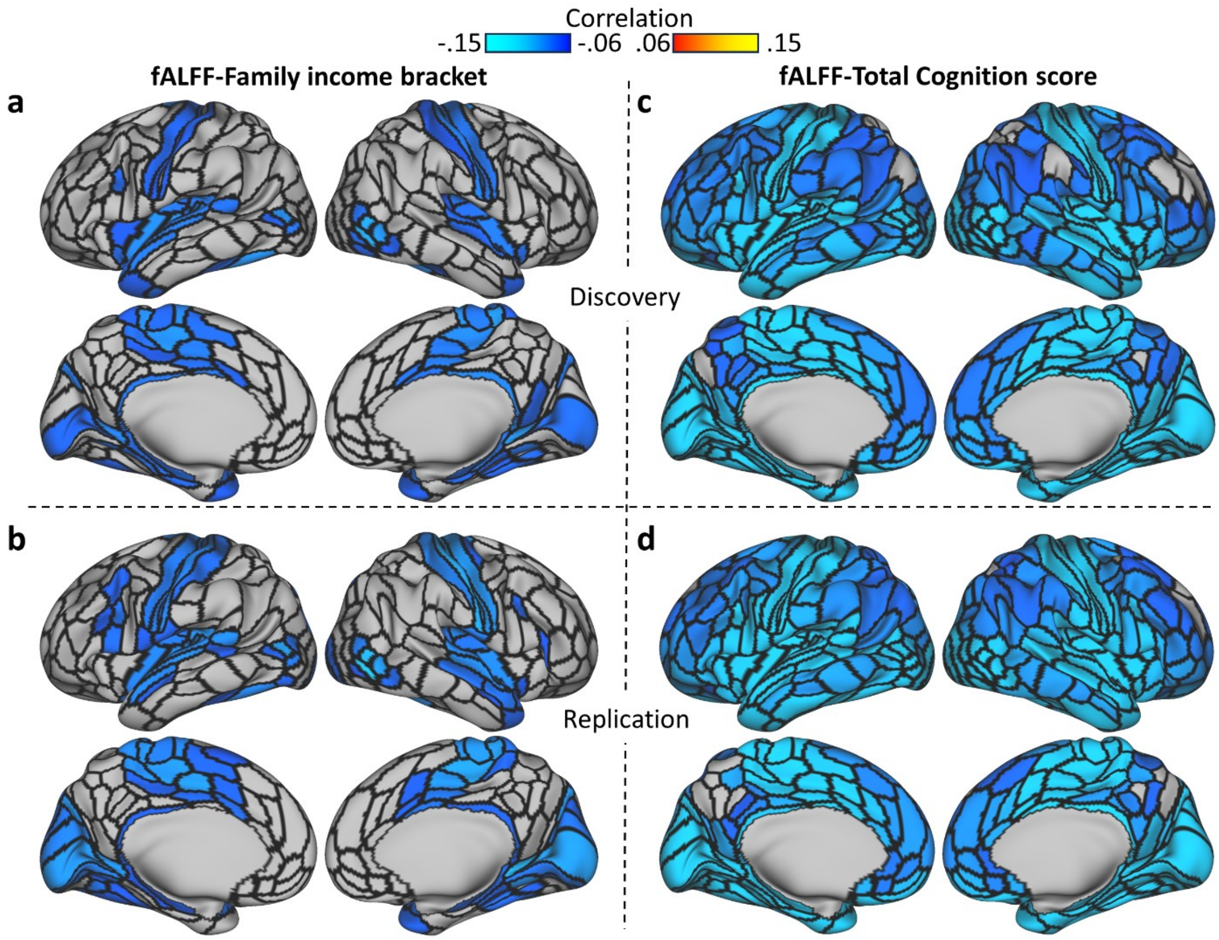

Supplemental Figure S8: Associations of with cognition and family income: fALFF. Higher family income bracket (**a-b**) and total cognition scores (**c-d**) were associated to lower bilateral fractional amplitude of low-frequency fluctuation (fALFF), independently in Discovery and Replication subsamples.

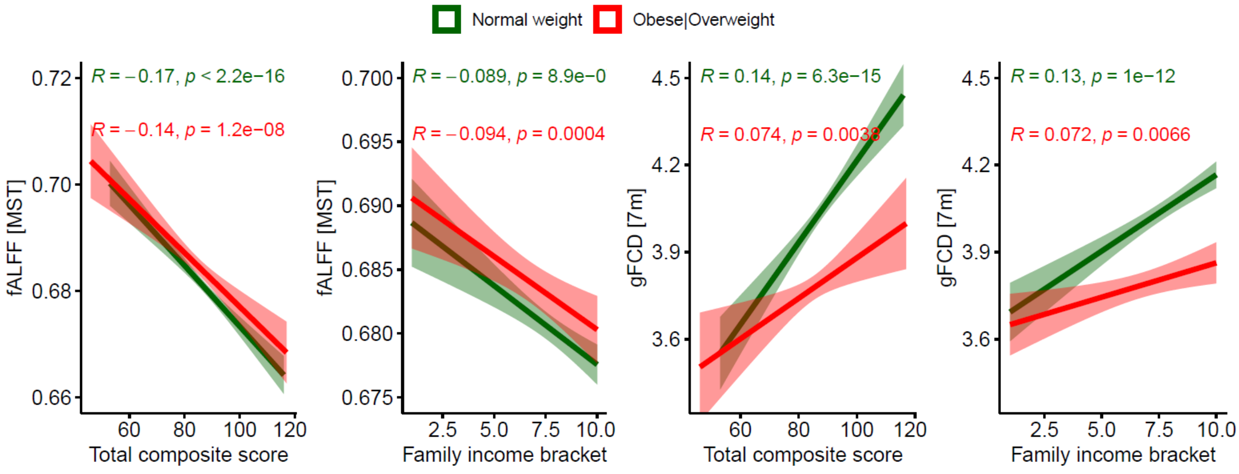

Supplemental Figure S9: Examples of linear associations with cognition and family income. Higher family income bracket and total cognition scores were associated to lower fractional amplitude of low-frequency fluctuation (fALFF) in the medial superior temporal (MST) area, and to higher global functional connectivity density (gFCD) in the precuneus area (7m), independently across normal weight and obese/overweight children.

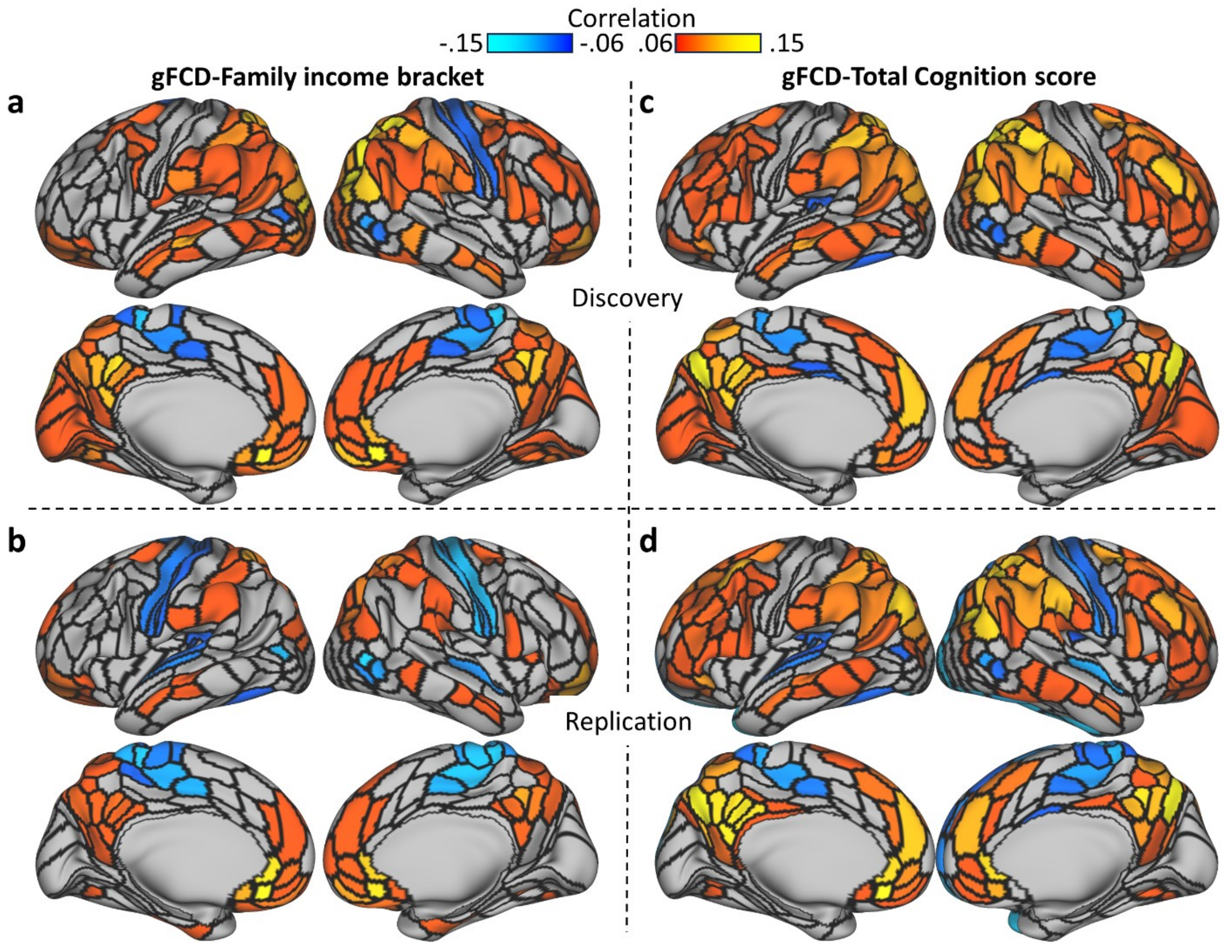

Supplemental Figure S10: Associations of with cognition and family income: gFCD. Associations between family income bracket (**a-b**) and total cognition scores (**c-d**) with global functional connectivity density (gFCD) overlaid on lateral and medial cerebral surfaces for the *Discovery* (n=3,597 children) and the *Replication* sample (n=3,513 children).

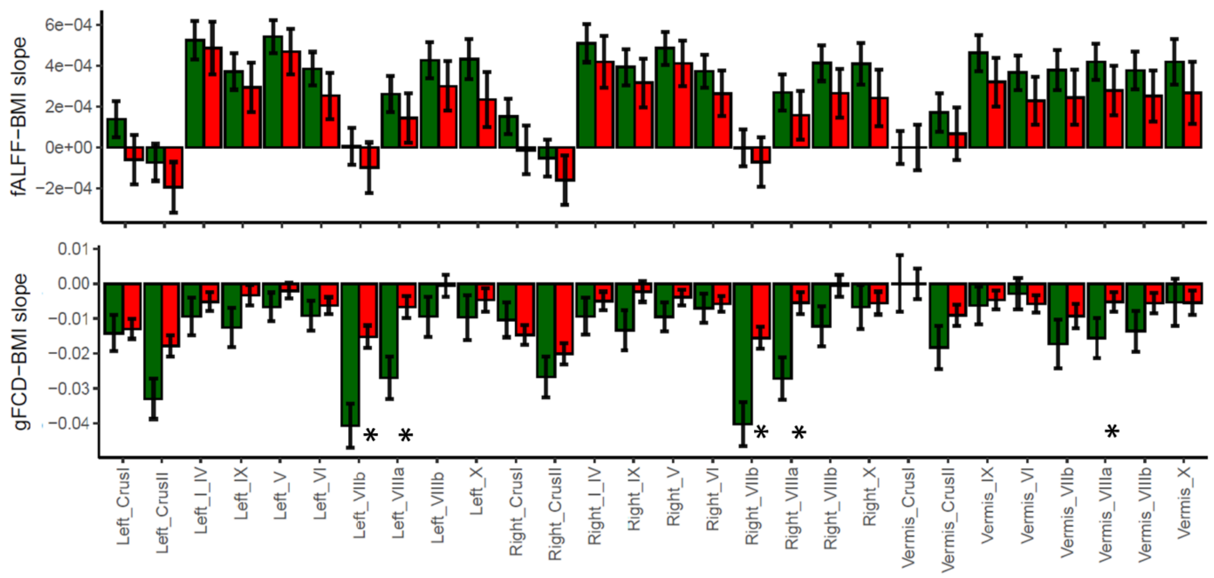

Supplemental Figure S11: Association with BMI in the cerebellum. Average slope of linear associations with BMI within 28 cerebellar partitions for fALFF and gFCD, independently for obese/overweight (n=2,356; red) and normal weight (n=4,754; green) children. *P-value < 0.001, uncorrected (two-sided t-test).

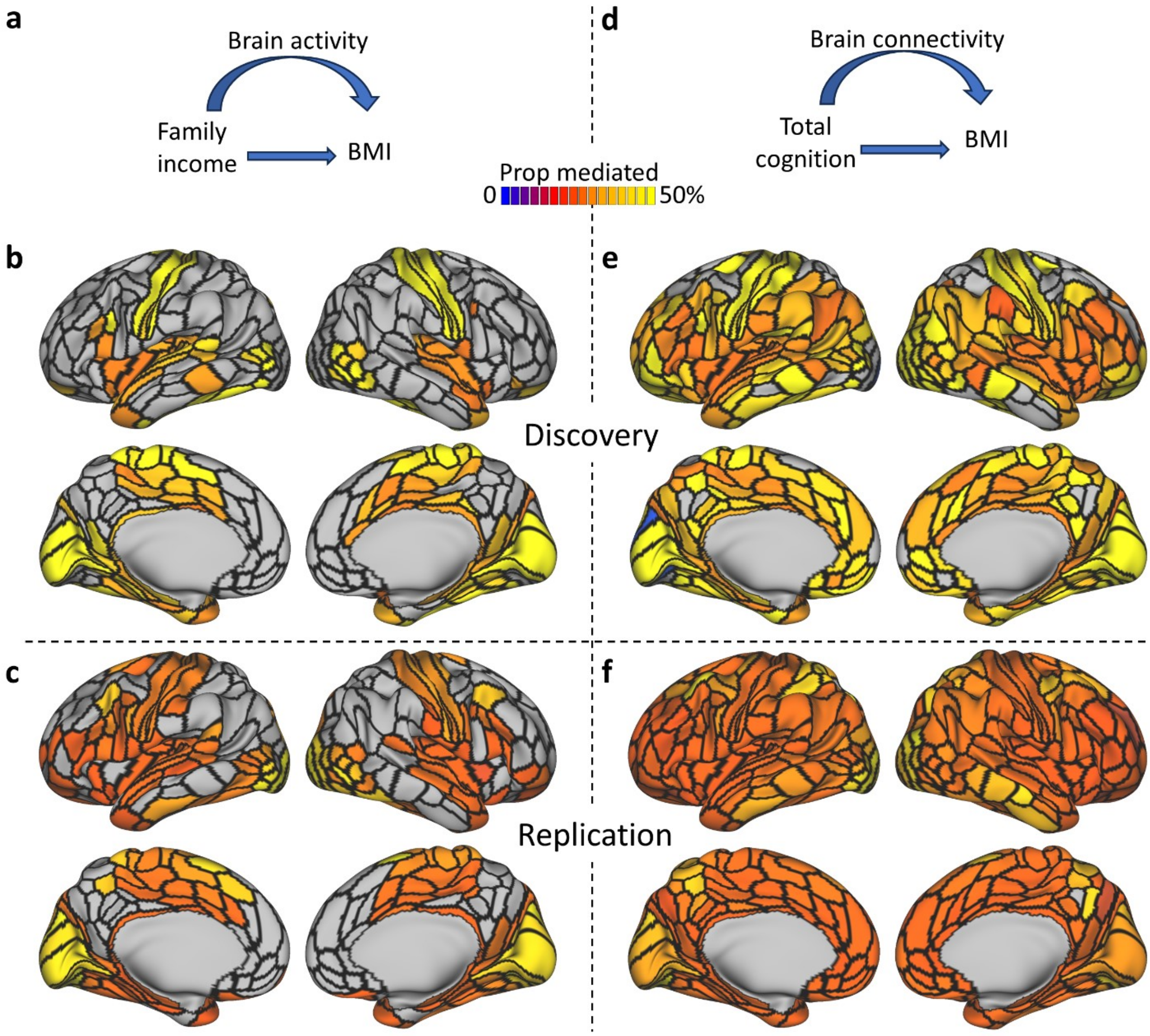

Supplemental Figure S12. Reproducibility of results from CMA model 1: fALFF. Proportion of the total effects of family income (**a-c**) or cognitive performance (**d-f**) on body mass index (BMI) that is mediated by the fractional amplitude of low-frequency fluctuations (fALFF) overlaid on lateral and medial cerebral surfaces for the Discovery (n=3,597 children) and the Replication sample (n=3,513 children). Threshold P_{ACME} <0.001.

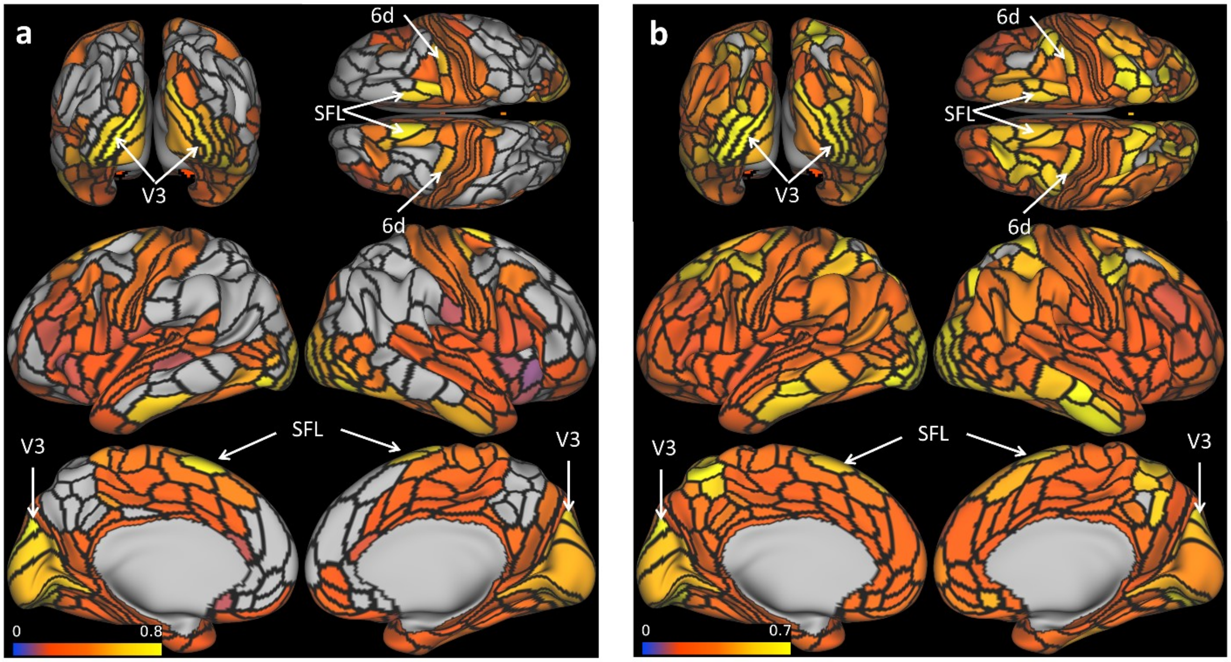

Supplemental Figure S13. Strongest mediators from CMA model 1: fALFF. Proportion of the total effects of family income (**a**) or cognitive performance (**b**) on body mass index (BMI) that is mediated by the fractional amplitude of low-frequency fluctuations (fALFF) across 7,110 children, overlaid on lateral and medial cerebral surfaces. Threshold P_{ACME} <0.001. Regions of interest with high mediation proportion: superior frontal language (SFL), premotor cortex (6d), and secondary visual cortex (V3) areas.

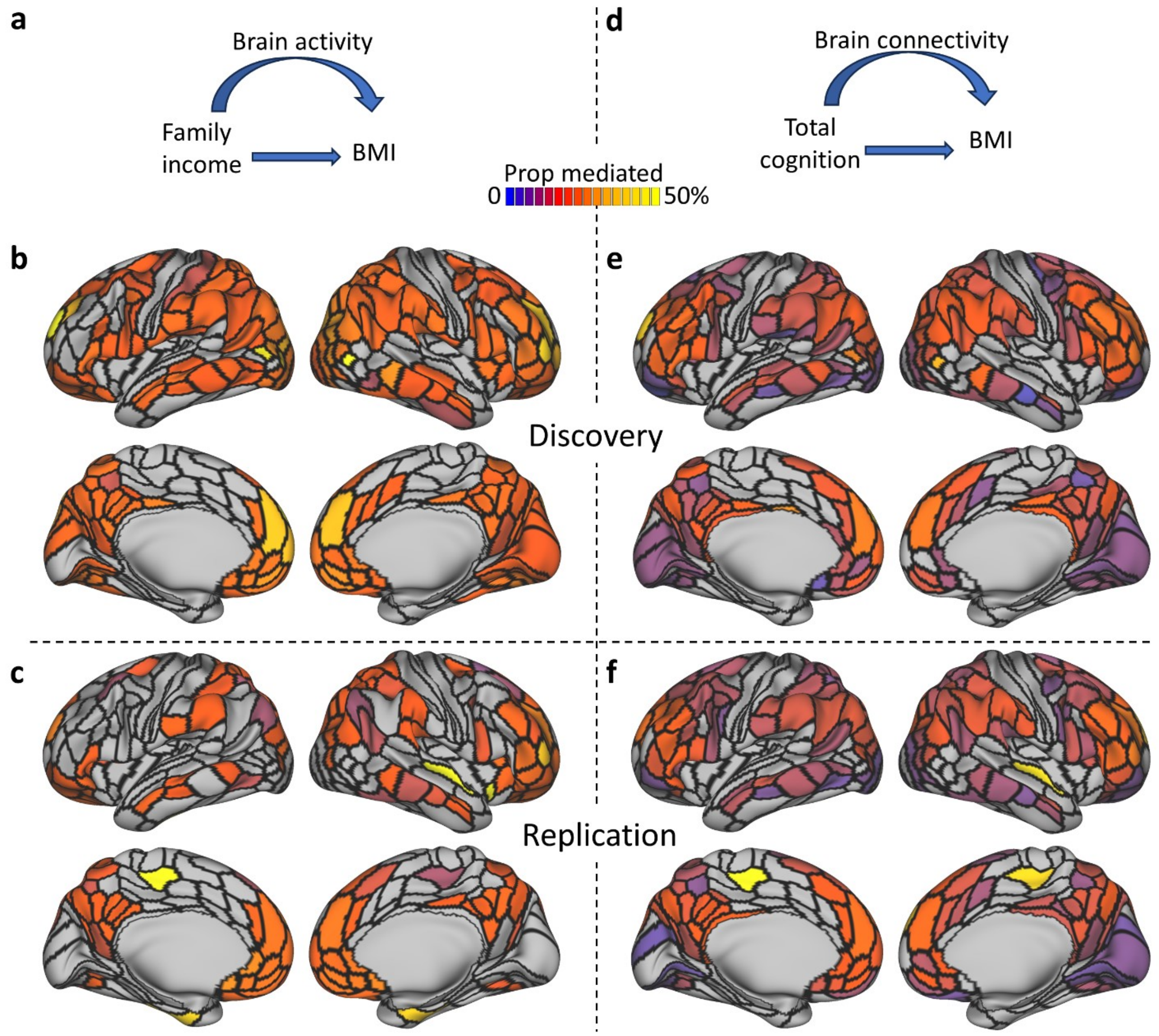

Supplemental Figure S14. Reproducibility of results from CMA model 1: gFCD. Proportion of the total effects of family income (**a**-**c**) or cognitive performance (**d**-**f**) on body mass index (BMI) that is mediated by the global functional connectivity density (gFCD) overlaid on lateral and medial cerebral surfaces for the *Discovery* (n=3,597 children) and the *Replication* sample (n=3,513 children). Threshold P_{ACME} <0.001.

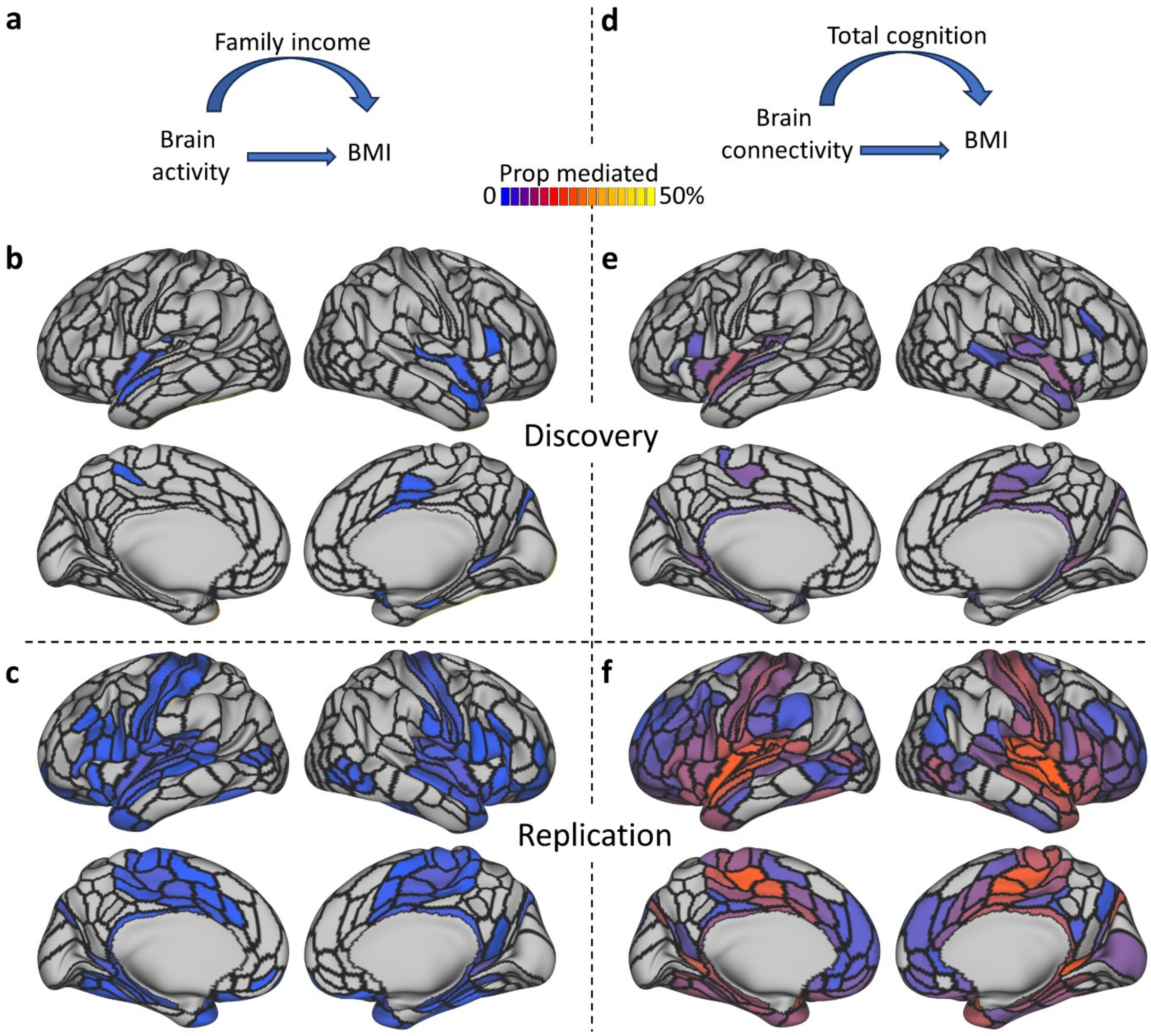

Supplemental Figure S15. Reproducibility of results from CMA model 2: fALFF. Proportion of the total effects of the fractional amplitude of low-frequency fluctuations (fALFF) on body mass index (BMI) that is mediated by family income (**a**-**c**) or cognitive performance (**d**-**f**) overlaid on lateral and medial cerebral surfaces for the *Discovery* (n=3,597 children) and the *Replication* sample (n=3,513 children). Threshold P_{ACME} <0.001.

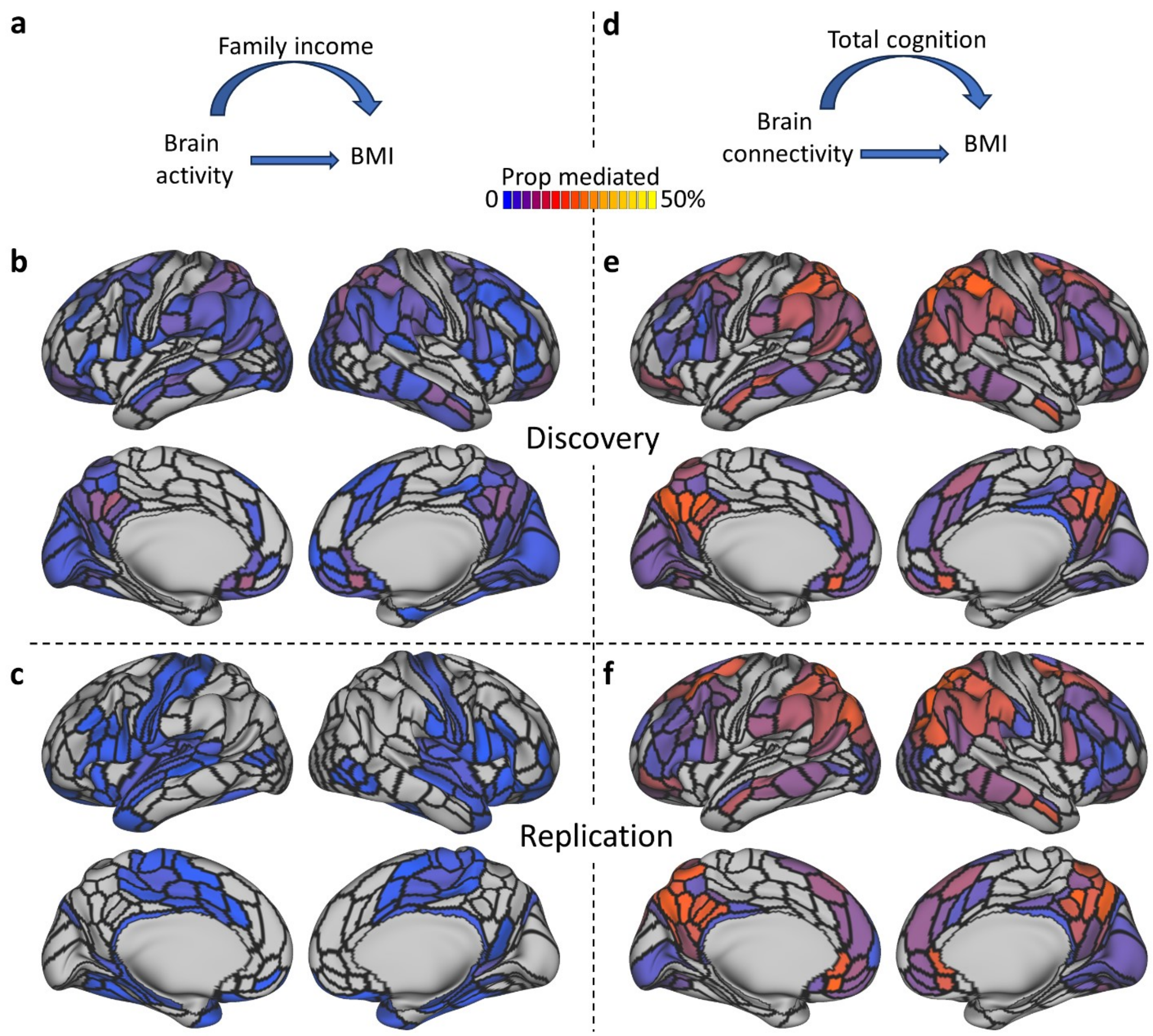

Supplemental Figure S16. Reproducibility of results from CMA model 2: gFCD. Proportion of the total effects of the global functional connectivity density (gFCD) on body mass index (BMI) that is mediated by family income (**a-c**) or cognitive performance (**d-f**) overlaid on lateral and medial cerebral surfaces for the *Discovery* (n=3,597 children) and the *Replication* sample (n=3,513 children). Threshold P_{ACME} <0.001.

Supplemental Table S1: Statistics for the main effects of family income bracket, total cognitive composite, and weight category (W; normal weight or obese/overweight) and their interactions (:) on the body mass index. ANCOVA model: BMI~ age*W + income*W + total*W.

	Discovery				Replication			
	Df	F	P	η^2	Df	F	P	η^2
**Age**	1	152	<2.2E-16	0.02	1	74	<2.2E-16	0.009
**W**	1	6,106	<2.2E-16	0.64	1	5,182	<2.2E-16	0.60
**Income**	1	50.7	1.3E-12	0.005	1	86	<2.2E-16	0.01
**Total**	1	4.0	0.04	<0.001	1	14	2.3E-04	0.002
**W: Age**	1	8.9	0.003	<0.001	1	14	2.0E-04	0.002
**W: income**	1	15.8	7E-05	0.002	1	54	2.0E-13	0.006
**W: total**	1	3.5	0.06	<0.001	1	8.1	0.004	0.001
**Residuals**	3,240				3.168			

Supplemental Table S2: Statistics for the main effects of family income bracket, total cognitive composite, body mass index (BMI) and their interaction (:) with weight category (W; underweight, normal weight, overweight, and obese) on the longitudinal diffusivity (lD) in the corpus callosum. ANCOVA model: lD~ age*W + BMI*W + income*W + total*W.

	**Df**	**F**	**P**	η^2
**Age**	1	25	6.3e-7	0.005
**W**	1	153	<2.2E-16	0.03
**BMI**	1	106	<2.2E-16	0.02
**Income**	1	18	1.8E-5	0.004
**Total**	1	9	0.003	0.002
**Age: W**	1	1	NS	0
**W: BMI**	1	1	NS	0
**W: income**	1	1	NS	0
**W: total**	1	1	NS	0
**Residuals**	4410			

References

1. Garavan H, Bartsch H, Conway K, Decastro A, Goldstein R, Heeringa S, et al. Recruiting the ABCD sample: Design considerations and procedures. *Dev Cogn Neurosci.* 2018;32:16-22.
2. Thompson W, Barch D, Bjork J, Gonzalez R, Nagel B, Nixon S, et al. The structure of cognition in 9 and 10 year-old children and associations with problem behaviors: Findings from the ABCD study's baseline neurocognitive battery. *Dev Cogn Neurosci.* 2019;36:100606.
3. Jernigan T, and Brown S. Introduction. *Dev Cogn Neurosci.* 2018;32:1-3.
4. Karcher N, Barch D, Avenevoli S, Savill M, Huber R, Simon T, et al. Assessment of the Prodromal Questionnaire-Brief Child Version for Measurement of Self-reported Psychoticlike Experiences in Childhood. *JAMA Psychiatry.* 2018;75(8):853-61.
5. Glasser M, Smith S, Marcus D, Andersson J, Auerbach E, Behrens T, et al. The Human Connectome Project's neuroimaging approach. *Nat Neurosci.* 2016;19(9):1175-87.
